# Development of a novel β-1,6-glucan–specific detection system using functionally-modified recombinant endo-β-1,6-glucanase

**DOI:** 10.1074/jbc.RA119.011851

**Published:** 2020-03-04

**Authors:** Daisuke Yamanaka, Kazushiro Takatsu, Masahiro Kimura, Muthulekha Swamydas, Hiroaki Ohnishi, Takashi Umeyama, Fumitaka Oyama, Michail S. Lionakis, Naohito Ohno

**Affiliations:** ‡ Laboratory for Immunopharmacology of Microbial Products, School of Pharmacy, Tokyo University of Pharmacy and Life Sciences, Hachioji, Tokyo 192-0392, Japan; § Fungal Pathogenesis Section, Laboratory of Clinical Immunology and Microbiology, NIAID, National Institutes of Health, Bethesda, Maryland 20892; ¶ Department of Chemistry and Life Science, Kogakuin University, Hachioji, Tokyo 192-0015, Japan; ‖ Research Fellow of Japan Society for the Promotion of Science (DC2), Koujimachi, Chiyoda-ku, Tokyo 102-0083, Japan; ** Department of Laboratory Medicine, Kyorin University School of Medicine, Mitaka, Tokyo 181-8611, Japan; ‡‡ Department of Chemotherapy and Mycoses, National Institute of Infectious Diseases, Shinjuku-ku, Tokyo 162-8640, Japan

**Keywords:** glycobiology, glycoside hydrolase, Candida albicans, animal model, infectious disease, fungi, β-1,3-D-glucan, β-1,6-glucan, deep mycosis, endo-β-1,6-glucanase

## Abstract

β-1,3-d-Glucan is a ubiquitous glucose polymer produced by plants, bacteria, and most fungi. It has been used as a diagnostic tool in patients with invasive mycoses via a highly-sensitive reagent consisting of the blood coagulation system of horseshoe crab. However, no method is currently available for measuring β-1,6-glucan, another primary β-glucan structure of fungal polysaccharides. Herein, we describe the development of an economical and highly-sensitive and specific assay for β-1,6-glucan using a modified recombinant endo-β-1,6-glucanase having diminished glucan hydrolase activity. The purified β-1,6-glucanase derivative bound to the β-1,6-glucan pustulan with a *K_D_* of 16.4 nm. We validated the specificity of this β-1,6-glucan probe by demonstrating its ability to detect cell wall β-1,6-glucan from both yeast and hyphal forms of the opportunistic fungal pathogen *Candida albicans*, without any detectable binding to glucan lacking the long β-1,6-glucan branch. We developed a sandwich ELISA-like assay with a low limit of quantification for pustulan (1.5 pg/ml), and we successfully employed this assay in the quantification of extracellular β-1,6-glucan released by >250 patient-derived strains of different *Candida* species (including *Candida auris*) in culture supernatant *in vitro*. We also used this assay to measure β-1,6-glucan *in vivo* in the serum and in several organs in a mouse model of systemic candidiasis. Our work describes a reliable method for β-1,6-glucan detection, which may prove useful for the diagnosis of invasive fungal infections.

## Introduction

β-Glucan is composed of d-glucose units linked by β-1,3-glycosidic bonds (β-1,3-d-glucan), which is the most common β-glucan structure produced by plants (β-1,3-/β-1,4-glucan) (1), bacteria (β-1,3-glucan) (2), fungi (3), and algae (4) (β-1,6-/β-1,3-glucan); of interest, β-1,3-d-glucan has been at the mainstream of glucan research. In addition, β-1,3-d-glucan–specific recognition proteins such as the limulus coagulation factor G in horseshoe crab (5), β-1,3-glucan recognition protein in insects (6), dectin-1 (7), and immunoglobulin (8, 9) in mammals are discovered in a wide range of species and applied to β-1,3-d-glucan–specific detection systems (10–13). Among these, the most commonly used in the world is factor G from horseshoe crab, a highly-sensitive and rapid assay.

The horseshoe crab (*Limulus polyphemus* and *Tachypleus tridentatus*)-derived *Limulus* amebocyte lysate (LAL)^2^ test has been evaluated since 1995 to detect β-1,3-d-glucan that is a marker of invasive fungal infections (14) and was approved by the United States Food and Drug Administration in 2004. However, false-negative or false-positive results were shown by the LAL test in some cases, because (i) not all pathogenic fungi release β-1,3-d-glucan, and (ii) plant- or bacteria-derived β-1,3-d-glucan leads to activation of limulus factor G unintentionally (15). Therefore, additional fungal diagnostic tests should be performed beyond β-1,3-d-glucan to accurately diagnose invasive fungal disease in the clinic.

One of the most common pathogenic fungal species, *Candida albicans*, releases a soluble mannoprotein–β-glucan complex that can activate limulus factor G (16). These glucan complexes can be detected by the LAL G test and in some cases have helped clinical decisions to start treatment early during fungal infection and to determine whether to administer antifungal or antibacterial drugs when an infection is suspected. Moreover, because first-line antifungal agents are different for each fungal species, diagnosing pathogenic fungal species early on by blood tests is important for a favorable outcome of patients. Interestingly, previous NMR analysis revealed that soluble extracellular polysaccharides of *C. albicans* cultured in the β-glucan–free medium were mainly composed of α-mannan and β-1,6-glucan, suggesting that the limulus factor G–reactive site (β-1,3-d-glucan) was a rather minor moiety (17). Although commercially-available diagnostic reagents targeting mannan have already been developed using a rabbit polyclonal antibody (CAND-TEC and UNIMEDI *Candida*) or a rat mAb (EBCA-1; PLATELIA *Candida* Ag and PASTREX *Candida*), there is no available tool for targeting the β-1,6-glucan structure thus far. Therefore, we hypothesized that if a tool to quantify β-1,6-glucan was developed, it would be useful to compensate for the shortcomings of the LAL test, and it may provide a potential avenue for future diagnostic test development in clinical practice.

In this study, we aimed to develop a new simple and convenient method for detection and quantification of β-1,6-glucan. To establish a new tool with high sensitivity at a low cost, certain conditions were required for probe candidates as follows: (i) high affinity and specificity for the β-1,6-glucan structure; (ii) a stable monomeric protein; and (iii) being efficiently produced by *Escherichia coli*. Among the different candidates, we focused on the endo-β-1,6-glucanase (EC 3.2.1.75), which is classified into glycoside hydrolase (GH) families 5 and 30 in the Carbohydrate-Active enZymes database (CAZy; RRID:SCR_012909). Several enzymes have been identified, cloned from fungi and bacteria, and further characterized for their structure-specific responses to β-1,6-glucan. Although natural glycoside hydrolases efficiently degrade polysaccharides, we hypothesized that the elimination of the hydrolytic activity of β-1,6-glucanase by a point mutation in the catalytic domain might still retain its glucan-binding activity. Because *Neurospora*-derived endo-β-1,6-glucanase (Neg1), which belongs to the GH family 30 subfamily 3, was well-characterized (18) and first successfully expressed in *E. coli* (19), we first attempted to evaluate modified enzymes based on Neg1. The putative catalytic residues for the acid/base and the nucleophile, the common catalytic glutamic acid residues (20–22) of GH family 30, which were also found in Neg1 (Glu-225 and Glu-321), were mutated to glutamine (Gln) to eliminate its hydrolase activity. We further characterized the glucan-binding capacity of this modified form of β-1,6-glucanase. Our results demonstrate that the modified recombinant β-1,6-glucanase retained its structure-specific glucan-binding activity, and thus, it can be employed as a novel β-1,6-glucan–specific detection probe.

## Results

### Point mutations in the catalytic domain of endo-β-1,6-glucanase promote its glucan-binding function

Pustulan is one of the most frequently-used soluble β-1,6-glucan standards. The LAL test did not show strong reactivity toward pustulan and other soluble β-1,6-glucans such as mushroom-derived AgCAS due to the presence of the extremely-low content of the β-1,3-glucan moiety in these forms. Instead, it recognized various β-1,3-glucans such as laminarin, single-strand SPG (schizophyllan), and pachyman (Figs. S1 and S2), especially pachyman showed ∼500 times stronger reactivity than pustulan (Fig. S2). Therefore, to enable specific detection of β-1,6-glucan structures, we aimed to develop specific probes by modifying the *Neurospora* endo-β-1,6-glucanase Neg1. We generated plasmids encoding the mature form of the Neg1 protein with point mutations at the catalytic positions (*i.e.* Glu-225, Glu-321, or both) to glutamine (Fig. S3*A*), and then the recombinant Neg1 and its variants were efficiently expressed in *E. coli* (Table S3). A single band with expected molecular weight for each of the purified proteins was detected (Fig. S3, *B* and *C*). Whereas Neg1 strongly hydrolyzed pustulan and produced oligosaccharides, including a range of glucose tetramers to monomers (Fig. S4*A*), and also increased the amount of reducing sugar in the reaction mixture (Fig. S4*B*), the glucanase derivatives Neg1–E225Q, Neg1–E321Q, and Neg1–E225Q/E321Q did not increase the reducing sugar (Fig. S4*B*).

**Figure 1.**
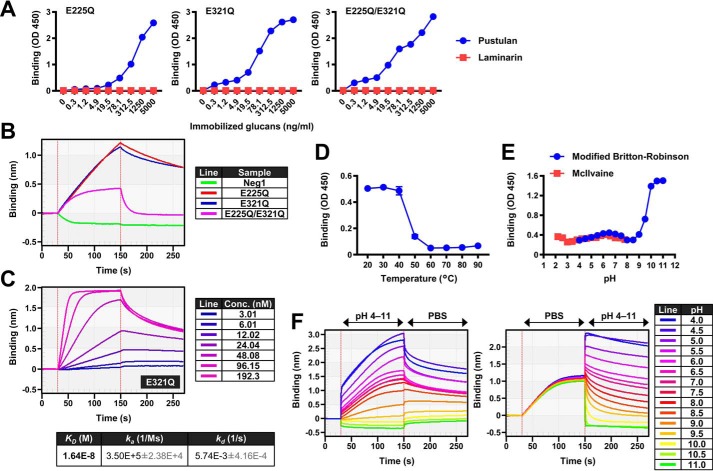
**β-1,6-Glucanase retains its ability to capture β-1,6-glucan after losing its glucan hydrolase activity via point mutations in the catalytic domain.** *A,* direct binding activity of Neg1 variants to pustulan. The binding capacity of β-1,6-glucanase variants Neg1–E225Q, Neg1–E321Q, and Neg1–E225Q/E321Q to solid-phased laminarin (*red*) or pustulan (*blue*) was evaluated by a direct ELISA-like assay. *B* and *C*, affinities of Neg1 variants to pustulan. The kinetic binding level of Neg1 and its variants to the pustulan-conjugated spencer tip was monitored by the BLI method (*B*), and the *K_D_* value of E321Q–His was calculated with 2-fold serially-diluted probes (*C*). *D,* thermal stability of E321Q–His. The binding activity of heat-treated (range, 20–90 °C for 5 min) E321Q–His to pustulan was verified with a direct ELISA-like assay using pustulan-coated plates. *E,* pH stability of E321Q–His. E321Q–His diluted in various pH conditions with McIlvaine (range, pH 2.2–7.8, *red*) or modified Britton-Robinson (range, pH 4–11, *blue*) buffer was incubated with solid-phased pustulan, and the glucan-binding capacity of E321Q–His was evaluated by direct ELISA. *F,* effect of pH on the glucan-binding ability of E321Q–His during association or dissociation by the BLI method. For analyzing the association phase, the pustulan-conjugated spencer tip was incubated with E321Q–His in assay buffer (pH 4–11) regulated with modified Britton-Robinson, and dissociation data were collected with PBS (*left panel*). For analyzing the dissociation phase, the spencer tip was incubated with E321Q–His in PBS, and the dissociation data were collected with assay buffer (pH 4–11) regulated with modified Britton-Robinson (*right panel*). Representative graphs from at least two independent experiments per assay are shown.

Next, to evaluate whether the glucan-binding activity was preserved in Neg1 with the different aforementioned point mutations, we carried out experiments with ELISA and bio-layer interferometry (BLI). As shown in Fig. 1*A*, all three variants showed binding to plate-coated pustulan, although they did not bind to immobilized β-1,3-glucan (laminarin). The Neg1–E321Q and Neg1–E225Q/E321Q variants in particular demonstrated greater reactivity even at lower concentrations (0.31–4.88 ng/ml) of solid-phased pustulan. The direct binding between β-1,6-glucan and Neg1 derivatives was then examined by BLI using a pustulan-conjugated sensor chip. Both Neg1–E225Q and Neg1–E321Q showed stronger binding activity to pustulan compared with Neg1–E225Q/E321Q (Fig. 1*B*). Interestingly, WT Neg1 could not retain itself on the polysaccharide due to the strong hydrolytic activity of the enzyme. We calculated the *K_D_* value of the binding of Neg1–E321Q to the pustulan immobilized on the biosensor using BLI because this variant showed strong binding to the β-1,6-glucan in both the ELISA and BLI tests, and the affinity (*K_D_* 1.64 × 10^−8^ m) showed a sufficient value for further investigation as a new glucan probe (Fig. 1*C*). This was further supported by the results of the isothermal titration calorimetry (ITC) analysis carried out for reference using the free unlabeled pustulan. The affinity was not very different from the one obtained by BLI, and we also confirmed that multiple proteins were bound to glucose polymers (binding ratio *n* = 0.14, 7.1:1 proteins/ligand).

Neg1–E321Q showed thermal stability up to 40 °C, whereas its binding function was completely abrogated when treated at 60 °C or higher for 5 min (Fig. 1*D*). Neg1–E321Q exhibited higher performance for ELISA at a neutral pH (pH 6–7) (Fig. 1*E*). Interestingly, the absorbance of ELISA was dramatically increased at a pH of 9 or greater (Fig. 1*E*); therefore, we further analyzed the effect of pH on the direct interaction of Neg1–E321Q with immobilized pustulan using the BLI method. During the association phase, strong binding was observed at pH values between 4.5 and 5.5, and decreased or absent binding was confirmed at pH values between 9 and 11 (Fig. 1*F*, *left panel*). Acidic conditions (pH values between 4.5 and 5.5) also improved the stability of Neg1–E321Q binding to glucan during the dissociation phase, and the dissociation rate became faster depending on the alkalinity of the test buffer (Fig. 1*F*, *right panel*).

The long-term stability of glutamine in Neg1–E321Q that was substituted in the catalytic domain of this enzyme was also quantitatively evaluated. This was done because, if glutamine reverts to glutamic acid over time, it would no longer function as a probe. The parental Neg1 exhibited the *K_m_* value of 1.1 ± 0.4 mg/ml for the increase of reducing sugars in natural substrates similar to a previous report (18). Moreover, the glucan hydrolase activity of Neg1–E321Q, which was stored for more than 2 years after purification, did not exhibit any *K_m_* value (Fig. S4*C*). We also proved that the modified Neg1–E321Q retained its sugar-binding activity (data not shown) and completely lost its glucan hydrolase activity upon long-term storage experiments for both natural and synthetic substrates (Fig. S4, *D–F*). Taken together, our data show that endo-β-1,6-glucanase exerts β-1,6-glucan–binding activity upon loss of its cleavage function via modifications of its catalytic site. This modified endo-β-1,6-glucanase exhibits stable activity even after long-term storage and thus showed promise for use as a novel β-1,6-glucan probe.

### Structure- and size-dependent binding of Neg1–E321Q to β-glucan

**Figure 2.**
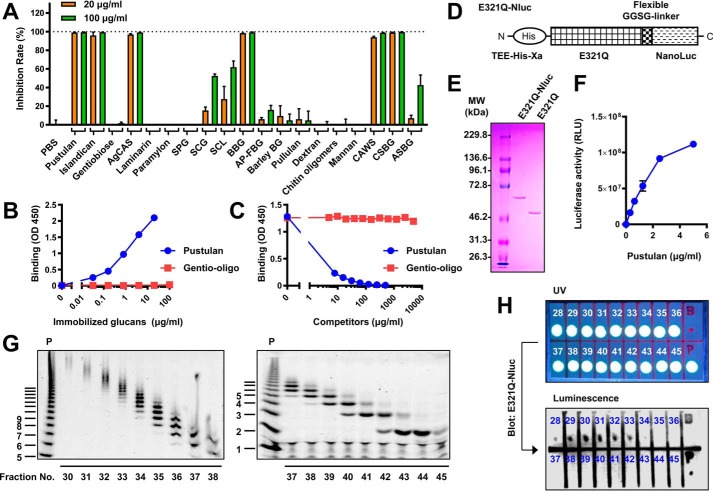
**Neg1–E321Q exerts structure-specific and molecular size-dependent ligand-binding activity.** *A,* reactivity of Neg1–E321Q with various glucans. Pustulan-coated plates were incubated with Neg1–E321Q in the presence of various glucans (concentration, 20 or 100 μg/ml) or PBS as a control. Data shown as inhibition rates (%) were calculated with absorbance from PBS as 0 and blank well as 100 and represent the mean ± S.D. of values of duplicate analyses. *B,* direct ELISA using pustulan- or gentio-oligosaccharides (DP 2–6)-coated plates and Neg1–E321Q. *C,* competitive ELISA was employed to verify the interaction between Neg1–E321Q and low molecular weight β-1,6-glucan. A pustulan-coated plate was incubated with Neg1–E321Q in the presence of soluble pustulan or gentio-oligosaccharides (DP 2–6). *D,* schematic of the Neg1–E321Q–Nluc fusion protein. *E,* SDS-PAGE image of E321Q–Nluc. Purified recombinant Neg1–E321Q–Nluc and Neg1–E321Q were separated using 11% polyacrylamide gel, and bands were visualized by Coomassie Brilliant Blue. *F,* direct binding of Neg1–E321Q–Nluc to β-1,6-glucan. Pustulan (concentration, 0–5 μg/ml)-coated plates were blocked and incubated with Neg1–E321Q–Nluc (2 μg/ml) for 1 h, and the luciferase activity was measured. Data represent the mean ± S.D. of values of triplicate analyses. *G,* HPLC separation of hydrolyzed pustulan and visualized by FACE. Pustulan was hydrolyzed with hydrochloric acid, lyophilized, and dissolved in water. Samples were fractionated every minute, labeled with ANTS, and analyzed by FACE using 30% (*left panel*) and 40% (*right panel*) gel. ANTS-labeled hydrolyzed pustulan (before HPLC separation) is shown in the *left margin* as standards (indicated as *P*). *H,* dot-blot analysis using Neg1–E321Q–Nluc. Each ANTS-labeled fraction of HPLC was spotted onto the membrane. The image was taken under UV light (*upper panel*) and by using a chemiluminescent scanner (*lower panel*). *Number* indicates each fraction. Blank sample (bromphenol blue) and ANTS-labeled hydrolyzed pustulan (before HPLC separation) were also spotted on the area labeled *B* and *P*, respectively. Shown are representative results from at least two independent experiments.

The structural specificity of the binding of Neg1–E321Q to the polysaccharide was then assessed by competitive ELISA using pustulan-coated plates. Soluble glucans mainly composed of β-1,6-glucan such as pustulan, islandican, and AgCAS strongly inhibited the binding between Neg1–E321Q and solid-phased pustulan (Fig. 2*A*). In contrast, linear β-1,3-glucan (*i.e.* paramylon) and soluble β-1,3-glucan with β-1,6-monoglycoside-branched side chains (*i.e.* laminarin and SPG) did not interfere with Neg1–E321Q binding. Binding of Neg1–E321Q to pustulan was also strongly inhibited by β-1,6-/β-1,3-complex glucan (*i.e.* SCG, *Sparassis* β-glucan; SCL, scleroglucan; and BBG, bakers' yeast-derived β-glucan), and it was only slightly inhibited by AP-FBG (*i.e.* β-1,3-glucan with β-1,6-glycoside highly-branched side chains) in a concentration-dependent manner. Moreover, extracellular polysaccharide from *C. albicans* (CAWS) and cell wall β-glucan from *C. albicans* (CSBG) and *Aspergillus* (ASBG) blocked the binding of Neg1–E321Q to pustulan. Instead, other glucans such as barley BG (*i.e.* β-1,3-/β-1,4-glucan), dextran (*i.e.* α-1,4-/α-1,6-glucan), pullulan (*i.e.* α-1,4-/α-1,6-glucan), and chitin oligomers and α-mannan (*i.e.* α-1,6-/α-1,2- and α-1,3-mannan) did not show dose-dependent inhibition of Neg1–E321Q binding.

We next aimed to understand how many β-1,6-glucose units are necessary for the binding to Neg1–E321Q. A previous study showed that another endo-β-1,6-glucanase (*i.e.* BT3312) from *Bacteroides thetaiotaomicron* was active on gentiotriose (degree of polymerization (DP) 3) but not on gentiobiose (DP 2) (21). Neg1 also produced glucose monomers and dimers by hydrolyzing pustulan (Fig. S4*A*), suggesting that it could be active on gentiotriose. As expected, Neg1–E321Q did not bind to gentiobiose (Fig. 2*A*); however, it also did not bind to immobilized gentio-oligo mix that contains glucose dimers to hexamers (Fig. S4*A*) in the direct ELISA-like assay (Fig. 2*B*). The result of the competitive ELISA also supported the above result because even high concentrations of gentio-oligo mix (5 mg/ml) could not inhibit the interaction between Neg1–E321Q and solid-phased pustulan (Fig. 2*C*). Therefore, we prepared oligosaccharides with larger molecular weights from pustulan by hydrolysis with acid and separation by HPLC. Glucose polymers in each fraction were analyzed by fluorophore-assisted carbohydrate electrophoresis (FACE) after fluorophore-labeling (Fig. 2*G*) and were used in the dot-blot assay. To avoid an excessive washing process in this assay, we further designed *Oplophorus* luciferase (NanoLuc)-fused Neg1–E321Q (Neg1–E321Q–Nluc) (Fig. 2*D*) and confirmed the ability of purified Neg1–E321Q–Nluc (Fig. 2*E* and Table S3) to bind β-1,6-glucan by ELISA (Fig. 2*F*). The fluorophore-conjugated negative-charge glucose polymer in each fraction was spotted on the positively-charged nylon membrane (Fig. 2*H*, *upper panel*), incubated with Neg1–E321Q–Nluc, and the luciferase activity was observed in the acid-degraded pustulan (P) and fraction numbers 28–34 (see Fig. 2*H*, *lower panel*). According to this result, the minimum unit of β-1,6-glucose polymer that can be recognized by Neg1–E321Q is DP 11–15 (*i.e.* the major bands in fraction no. 34). Collectively, these results suggest that Neg1–E321Q has a strong structural specificity and molecular size dependence for binding to polysaccharides.

### Applying the β-1,6-glucanase Neg1–E321Q to the quantification of Candida β-1,6-glucan

**Figure 3.**
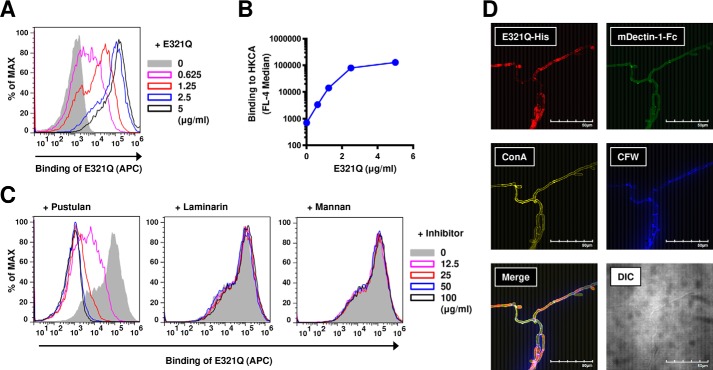
**Neg1–E321Q recognizes β-1,6-glucan on the cell wall of both yeast and hyphae forms of *C. albicans*.** *A* and *B*, direct binding of Neg1–E321Q–His (0–5 μg/ml) to the heat-killed yeast form of *C. albicans* (*HKCA*) was analyzed by FACS, and data are presented as representative histograms (*A*) and summary data of median fluorescence intensity (*B*). *C,* Structure-specific binding of Neg1–E321Q–His onto the yeast cell surface. HKCA was incubated with Neg1–E321Q–His in the presence of pustulan, laminarin, or mannan (0–100 μg/ml) and analyzed by FACS, and data are presented as representative histograms. *D,* Neg1–E321Q binds to the cell wall of the hyphal form of *C. albicans*. Fixed hyphae were stained with Neg1–E321Q–His and probes specific for β-1,3-glucan (dectin-1), mannan (ConA), and chitin (CFW). Shown are merged images from the four different probes that indicate localization. Differential interface contrast images are also shown. *Scale bars*, 50 μm. Shown are representative results from at least two independent experiments.

**Figure 4.**
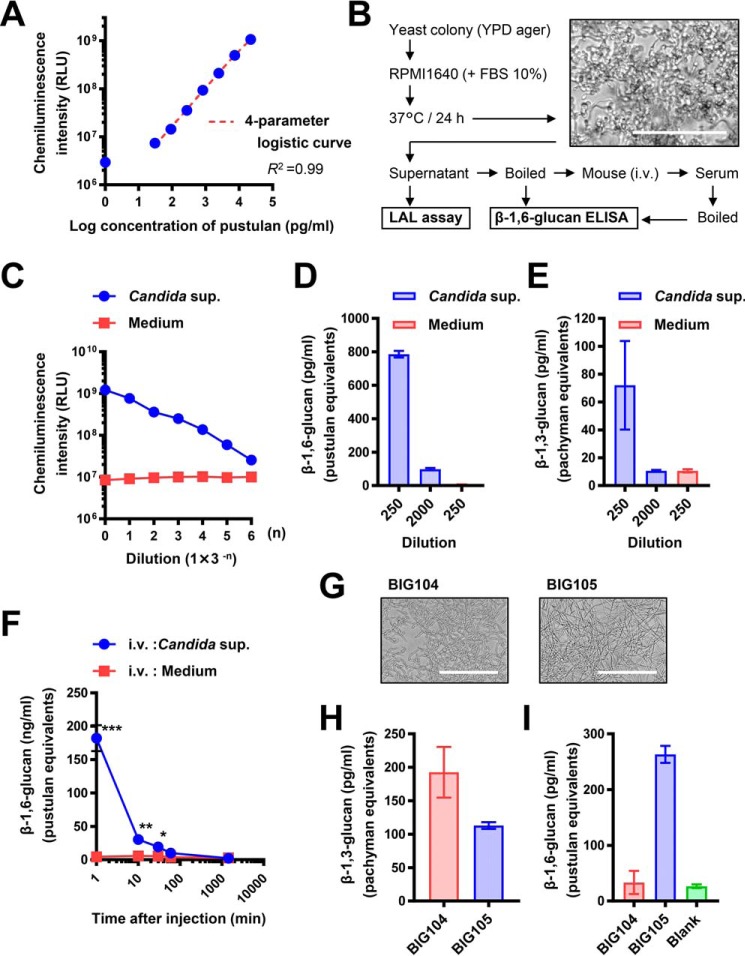
**Application of sandwich ELISA using Neg1–E321Q for the quantification of naturally released β-1,6-glucan from *C. albicans*.** *A,* standard curve of 3-fold serial dilutions of pustulan (concentration range, 30.5 pg/ml to 22.2 ng/ml). *B,* flowchart of the experiment for *in vitro* culture of *C. albicans* strain NBRC1385 and image of growth conformation. *C,* reactivity of sandwich ELISA to 3-fold serial dilutions of *C. albicans* strain NBRC1385 culture supernatant (*blue*) or *Candida*-free medium (*red*). β-1,6-Glucan (*D*) or β-1,3-glucan (*E*) content in culture supernatant of *C. albicans* strain NBRC1385 diluted 250- or 2,000-fold (*blue*) or *Candida*-free medium (*red*) is shown. *F,* blood clearance of β-1,6-glucan in mice. Serum was collected at 1, 10, and 30 min and at 24 h after intravenous injection of *C. albicans* NBRC1385 culture supernatant (*blue*) or *Candida*-free medium (*red*). The serum was diluted twice, and β-1,6-glucan concentrations were measured. *G,* images showing the proliferation of *C. albicans* strains BIG104 and BIG105. β-1,3-Glucan (*H*) or β-1,6-glucan (*I*) content in culture supernatants of *C. albicans* strains BIG104 and BIG105 or *Candida*-free medium is shown. Supernatants and blanks were diluted 100- and 50-fold for the β-1,6-glucan ELISA and LAL test, respectively. Data are presented as mean ± S.D. of values in duplicate (*A*, *C*, *D*, and *H*) or triplicate (*E* and *I*), or mean ± S.E. (*n* = 3) (*F*). Shown are representative results from at least two independent experiments. *Scale bars*, 100 μm.

Because Neg1–E321Q reacted with pathogenic fungus–related polysaccharides, particularly with the *Candida* cell wall (CSBG) and extracellular (CAWS) glucan (Fig. 2*A*), we next aimed to employ the β-1,6-glucanase assay for a potential diagnostic application. First, to demonstrate whether there is direct interaction between Neg1–E321Q and the cell surface of *C. albicans*, we carried out flow cytometric (for the yeast form) and microscopic (for the hyphal form) analyses using the *C. albicans* strain NBRC1385. Notably, Neg1–E321Q bound to the cell wall of the yeast form of *C. albicans* in a dose-dependent manner (Fig. 3, *A* and *B*), and this binding was clearly inhibited by the addition of soluble β-1,6-glucan, but not of β-1,3-glucan or mannan (Fig. 3*C*); this finding indicates that the binding between Neg1–E321Q and the yeast cell surface is mediated in a β-1,6-glucan–specific manner. In addition, Neg1–E321Q bound to the hyphal form of *C. albicans*. Of interest, the hyphal areas recognized by Neg1–E321Q were somewhat different from those recognized by dectin-1–Fc that stains β-1,3-glucan, concanavalin A that stains mannan, and calcofluor white that stains chitin (Fig. 3*D*). Next, to quantify the extracellular polysaccharides released from *C. albicans*, we prepared biotin-labeled Neg1–E321Q and assembled a sandwich ELISA. By comparison of the horseradish peroxidase (HRP) substrate, we applied both colorimetric and chemiluminescent methods using unlabeled Neg1–E321Q-coated microplates and biotin-labeled Neg1–E321Q with streptavidin–HRP and found reactivity to pustulan concentrations ranging from 1.4 to 1,000 pg/ml (Fig. S5, *A* and *B*). The limit of quantification of the colorimetric and chemiluminescent methods was 32.1 and 1.5 pg/ml, respectively (Fig. S5, *C* and *D*). Accordingly, we decided to use the chemiluminescent method for our subsequent experiments. The standard curve from a broad range of pustulan concentrations (*i.e.* 30.5 pg/ml to 22.2 ng/ml) is shown in Fig. 4*A*. Then, yeast colonies of *C. albicans* strain NBRC1385 were inoculated in RPMI 1640 medium containing 10% FBS and cultured at 37 °C for 24 h for the hyphal form to develop in order to measure the naturally-produced extracellular polysaccharides by *Candida* hyphae (Fig. 4*B*). The supernatants of the culture medium with or without *Candida* were then tested by sandwich ELISA, which showed reactivity to the *Candida* supernatant, but not to the *Candida*-free medium, in a dilution-dependent manner (Fig. 4*C*). Furthermore, the diluted supernatant was measured by both the β-1,6-glucan ELISA and the β-1,3-d-glucan LAL test. Notably, the Neg1–E321Q sandwich ELISA test could measure β-1,6-glucan in both 250- and 2,000-fold diluted *Candida* supernatants (Fig. 4*D*). Although the 250-fold diluted *Candida* supernatant also contained detectable β-1,3-d-glucan, the 2,000-fold diluted *Candida* supernatant only contained measurable β-1,6-glucan but not β-1,3-d-glucan (Fig. 4*E*).

Because we found that that extracellular polysaccharides could be detected in the culture supernatant even at a 2,000-fold dilution, that culture supernatant was then injected intravenously into mice to determine whether these circulating polysaccharides could be detected *in vivo* in mouse blood. After 1, 10, and 30 min of injection, we detected β-1,6-glucan in the serum by the sandwich ELISA using Neg1–E321Q. Instead, β-1,6-glucan was not detected in blood by 24 h after administration (Fig. 4*F*). Importantly, we did not detect a nonspecific signal in the serum of mice injected with the *Candida*-free medium.

To exclude the possibility that our ELISA might measure metabolic products other than β-1,6-glucan that could be released by *C. albicans*, we examined the *C. albicans* Ca*big1*Δ strain BIG104, which is known to have impaired β-1,6-glucan biosynthesis, together with the reconstituted *C. albicans* strain BIG105 that has intact β-1,6-glucan biosynthesis. The two strains grew similarly (Fig. 4*G*), and both produced β-1,3-glucan as measured by the LAL test (Fig. 4*H*). Instead, when we measured β-1,6-glucan in the supernatant of both strains by the sandwich ELISA using Neg1–E321Q, we detected β-1,6-glucan only in the reconstituted *C. albicans* strain BIG105, but not in the β-1,6-glucan–deficient *C. albicans* Ca*big1*Δ strain BIG104 (Fig. 4*I*). Together, this finding indicates that our sandwich ELISA specifically detects β-1,6-glucan, even within a crude biological specimen derived from *C. albicans*.

### β-1,6-Glucan is detected in the serum and tissue homogenates of Candida-infected mice by an ELISA-like assay

**Figure 5.**
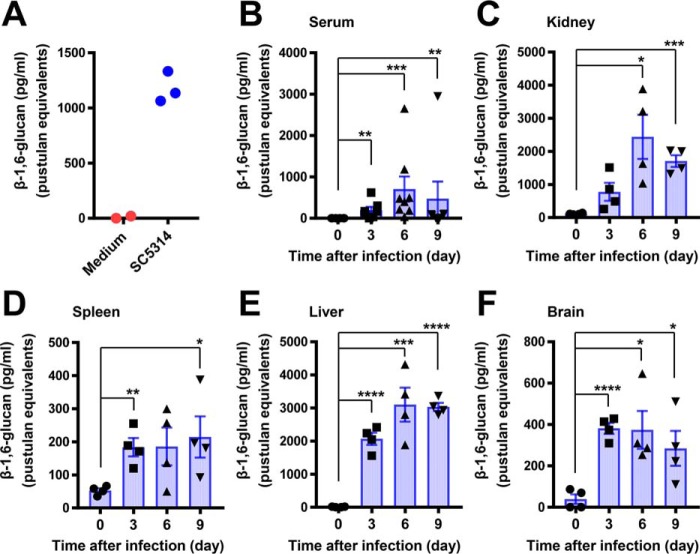
**β-1,6-Glucan is produced and can be detected in C57BL/6 mice after systemic *Candida* infection.** *A,* β-1,6-glucan production by *C. albicans* SC5314 *in vitro*. β-1,6-Glucan was measured in the *C. albicans* SC5314 culture supernatant after 24 h or in *Candida*-free medium as control, which were diluted 50-fold. An ELISA-like assay based on Neg1–E321Q with pustulan as the standard of β-1,6-glucan was used. *B–F,* β-1,6-glucan production by *C. albicans* SC5314 *in vivo*. Concentrations of β-1,6-glucan in serum (*B*), kidney (*C*), spleen (*D*), liver *(E*), and brain (*F*) isolated from C57BL/6 mice on days 0, 3, 6, and 9 after *C. albicans* intravenous injection were measured by sandwich ELISA. Data are presented as mean ± S.E. (*n* = 6–8 for serum and *n* = 4 for organ homogenates). Significant differences of days 3, 6, and 9 relative to day 0: *, *p* < 0.05; **, *p* < 0.01; ***, *p* < 0.001; ****, *p* < 0.0001.

We next wondered whether our sandwich ELISA could detect naturally-produced β-1,6-glucan *in vivo* in mice infected systemically with the *C. albicans* strain SC5314. First, we confirmed abundant production of β-1,6-glucan following *in vitro* culture of *C. albicans* strain SC5314 by ELISA (Fig. 5*A*). Next, we measured the concentration of β-1,6-glucan in the serum and homogenized kidney, spleen, liver, and brain of WT mice at days 3, 6, and 9 post-infection with *C. albicans* SC5314, and we found significant increases compared with uninfected control mice; the β-1,6-glucan concentration peaked at day 6 after infection at the peak of fungal proliferation in the model (Fig. 5, *B–F*) (23). We also examined β-1,6-glucan levels in *Candida*-infected Cx3cr1-deficient mice that exhibit greater tissue fungal burden and mortality relative to WT mice (24); the β-1,6-glucan content in serum and tissues of Cx3cr1-deficient mice tended to be higher than that of WT mice (Fig. S6). Together, these data show that β-1,6-glucan is produced *in vivo* in blood and various organs of *Candida*-infected mice and its temporal kinetics can be measured by our ELISA using Neg1–E321Q.

### β-1,6-Glucan is produced by a large number of clinical Candida strains irrespective of species

**Figure 6.**
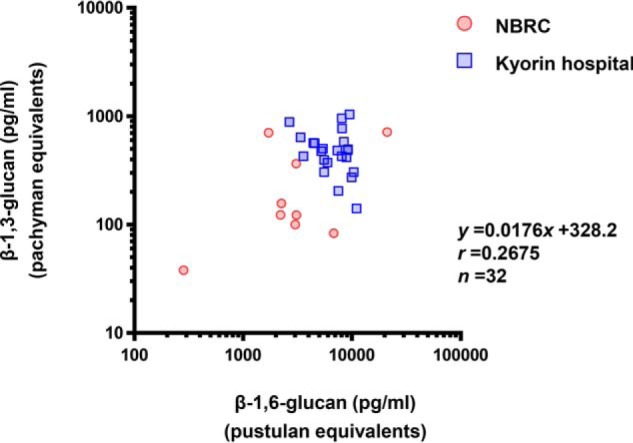
**Correlation between the detected concentrations of β-1,3-glucan and β-1,6-glucan in the culture supernatants of *C. albicans* isolates.** *C. albicans* yeasts obtained from NBRC (*n* = 9, *red*) or the Kyorin University Hospital (*n* = 23, *blue*) were cultured for 24 h *in vitro,* and the supernatant was diluted 10-fold. The β-glucan contents in the culture supernatants were measured by ELISA (for β-1,6-glucan) and the LAL test (for β-1,3-glucan). Pustulan and pachyman were used as the standard glucans for the ELISA and LAL test, respectively.

We have thus far have shown that β-1,6-glucan can be detected in the culture supernatants of three strains of *C. albicans* (*i.e.* NBRC1385, BIG105, and SC5314) by a sandwich ELISA using Neg1–E321Q. Because no information exists with regard to the ability of all *C. albicans* strains to produce β-1,6-glucan, we next examined levels of β-1,6-glucan (and of β-1,3-d-glucan as control) in 32 strains of *C. albicans* obtained from NITE Biological Resource Center (NBRC) (*n* = 9) and the Kyorin University Hospital (*n* = 23). The strains were cultured for 24 h *in vitro* and analyzed using both our ELISA method and the LAL test. As shown in Fig. 6, both β-1,6-glucan and β-1,3-d-glucan were detected in all tested *C. albicans* isolates, and we found a positive correlation between β-1,6-glucan and β-1,3-d-glucan levels in the tested strains.

**Figure 7.**
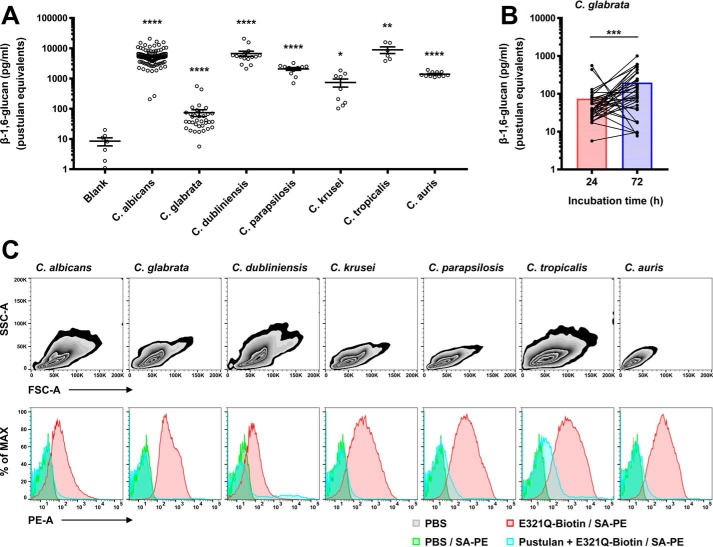
**β-1,6-Glucan is produced by and can be detected in a large number of clinical isolates of all major *Candida* species.** *A,* β-1,6-glucan contents in the culture supernatants of *C. albicans* and non-*albicans Candida* strains. Yeasts (*C. albicans,* 132; *C. glabrata,* 35; *C. dubliniensis,* 15; *C. parapsilosis,* 11; *C. krusei,* 11; *C. tropicalis,* 9; *C. auris,* 11) isolated at the National Institutes of Health Clinical Center and provided from the CDC AR bank were cultured for 24 h, and 10-fold diluted supernatants were analyzed using the β-1,6-glucan ELISA. *B,* kinetic production of β-1,6-glucan from *C. glabrata*. 35 strains of *C. glabrata* were cultured for 24 or 72 h, and β-1,6-glucan in 10-fold diluted supernatants was measured. *Bars* are presented as mean of values. *C.* β-1,6-glucan on the cell surface of *Candida* yeasts. Representative clinical isolates of the corresponding *Candida* species were fixed and incubated with PBS or Neg1–E321Q–biotin in the presence or absence of pustulan. Yeasts were further stained with PBS (control) or streptavidin-PE (*SA-PE*) and analyzed using flow cytometry. Significant differences from blank (*A*) or between two groups (*B*): *, *p* < 0.05; **, *p* < 0.01; ***, *p* < 0.001; and ****, *p* < 0.0001.

We next expanded our investigation in 224 other *Candida* clinical isolates across all *Candida* species to determine the extent and strain specificity of β-1,6-glucan production. For that, 132 *C. albicans* and 92 non-*C. albicans Candida* species (*Candida glabrata* (*n* = 35), *Candida dubliniensis* (*n* = 15), *Candida parapsilosis* (*n* = 11), *Candida krusei* (*n* = 11), *Candida auris* (*n* = 11), and *Candida tropicalis* (*n* = 9)) were cultured for 24 h, and the level of β-1,6-glucan production was measured for each strain by our ELISA. Notably, β-1,6-glucan was detected at high levels in the culture supernatants of all tested strains of *C. albicans*, *C. dubliniensis*, *C. parapsilosis*, *C. tropicalis,* and *C. auris* (Fig. 7*A*). *C. krusei* strains released β-1,6-glucan to a lower extent relative to the aforementioned *Candida* species. *C. glabrata* produced the least amount of β-1,6-glucan relative to all other *Candida* species; yet, the β-1,6-glucan measured in *C. glabrata* supernatants was significantly greater compared with that in the *Candida*-free medium. Because the growth rate of *C. glabrata* can be slower than that of other *Candida* species, we extended its incubation period to 72 h and observed a slight, yet significant, increase in β-1,6-glucan production in the culture medium in most tested *C. glabrata* strains (Fig. 7*B*).

Our data revealed that all *Candida* species produce β-1,6-glucan, but the extent of the production varies in different *Candida* species. We wondered whether the amount of β-1,6-glucan exposed on the cell wall of various *Candida* species might mirror the species-specific β-1,6-glucan production. For that, we employed FACS and used Neg1–E321Q as the probe to bind to yeast forms of representative strains from the seven different *Candida* species (Fig. 7*C*). This binding was specific as it was inhibited by the addition of pustulan. All tested strains from the seven *Candida* species isolated from patients had detectable β-1,6-glucan on their cell wall using this approach. Taken together, our data show that β-1,6-glucan can be produced and detected by our Neg1–E321Q–based ELISA in >250 clinical isolates of various *Candida* species. This finding together with the ability to detect β-1,6-glucan *in vivo* in the mouse model of systemic candidiasis provides the foundation for the future development and testing of β-1,6-glucan as a potentially useful diagnostic test in humans with invasive candidiasis.

## Discussion

In recent years, invasive fungal infections such as candidiasis and aspergillosis have emerged as important causes of morbidity and mortality in acutely ill patients in the intensive care unit and in immunosuppressed patients with cancer, and hematopoietic stem cell or solid organ transplantation. Mortality in patients affected by invasive fungal infections remains unacceptably high (>40%) despite administration of potent antifungal therapy (25, 26). A major cause for the high mortality in these patients is the delayed initiation of antifungal treatment, which is caused by the suboptimal performance of current diagnostic tests for invasive fungal infections (27). Specifically, fungal isolation and identification by culture and/or histopathological examination are hampered by low sensitivity, and even when positive, it typically takes several days to identify the infecting pathogen. PCR testing appears sensitive, but it is not standardized for clinical use (27). The recent advent of serological tests that measure fungal polysaccharides such as β-1,3-d-glucan and galactomannan has improved diagnostic accuracy in certain settings but still has limitations (27). Therefore, new diagnostic tests are needed to facilitate timely diagnosis of invasive fungal infections and improve patient outcomes.

Besides its decreased sensitivity and specificity, another important limitation of the LAL test that measures β-1,3-d-glucan is a large decline in the horseshoe crab population due to commercial harvesting. As such, although the LAL C test kit for measuring endotoxin has been reconstructed by animal-free recombinant proteins (28) (for example, PyroGene rFC/Lonza and PyroSmart/Seikagaku Corp.), the LAL G test that measures β-1,3-d-glucan is still made from blue blood collected from living horseshoe crabs.

In this study, we developed a β-1,6-glucan detection system with an animal-free recombinant protein. Before focusing on β-1,6-glucanase, we had considered other candidates for a β-1,6-glucan probe, such as the *Musa acuminata*–derived lectin (29), yeast-derived K1/K2 killer toxins (30), and a mAb (31). However, we excluded these candidates for the following reasons: the lectin has insufficient structure specificity; killer toxins are structurally unstable; and a mAb requires high cost for sufficient production in high-quality grade. As expected, *Neurospora* β-1,6-glucanase–derived genetically-engineered enzymes were efficiently expressed in *E. coli* (Table S3) without any refolding process, and they also exhibited the expected binding capacity for pustulan and various β-1,6-glucans. Moreover, they could easily be fused with other small proteins like *Oplophorus* luciferase (NanoLuc; Fig. 2*D*), and these modified enzymes were also found to exhibit stable glucan-binding activity without regaining glycolytic function even after long-term storage. For these reasons, we propose that the modified β-1,6-glucanase has a potential application and can be utilized in new fields. A future direction of research could focus on further enhancing the design of improved probes based on modifying the glucanase with a higher affinity to glucan.

A potential advantage of detecting β-1,6-glucan during infection is that it does not respond to β-glucans from plant (β-1,3-/β-1,4-glucan) or bacteria (β-1,3-glucan). In addition, our β-1,6-glucanase derivative did not respond to gentio-oligosaccharides (DP 2–10), a finding that suggests that low molecular weight oligos derived from food additives or botanical glycosides, like a crocin in saffron (*Crocus sativus*) (32), should not produce nonspecific reactions in the β-1,6-glucan test. Furthermore, the use of an immunoassay is facile. Indeed, β-1,6-glucan released from cultured *Candida* strains could be easily detected by our ELISA method. The absence of nonspecific reactivity of our modified β-1,6-glucanase to *Candida*-produced exopolysaccharide was proven by our analysis of *C. albicans* strain BIG104, which lacks β-1,6-glucan biosynthesis. Interestingly, the amount of released β-1,6-glucan varied depending on the *Candida* species, but it was detected in all medically important *Candida* species, with higher levels in *C. albicans*, *C. dubliniensis*, *C. parapsilosis*, and *C. tropicalis*; intermediate levels in *C. krusei* and *C. auris*; and lower levels in *C. glabrata*. The presence of β-1,6-glucan in the cell wall of *C. glabrata* was confirmed by our FACS results and is consistent with a previous report (33); however, the production level of extracellular β-1,6-glucan was lower in *C. glabrata*. Although the growth rate may have contributed to this lower production of β-1,6-glucan, other factors such as the greater evolutionary distance on the phylogenetic tree may also be operative (34). To clarify how much β-1,6-glucan is contained in the naturally-released exopolysaccharides from major pathogenic fungi of humans, including *Aspergillus* spp., *Mucorales* spp., *Cryptococcus* spp., and *Pneumocystis* spp., further investigation will be required in the future.

In developing a serum diagnostic method, it is necessary to consider the biological metabolism rate of the target molecule. The pharmacokinetic information pertaining to *C. albicans* cell wall β-glucan has previously been reported (35), and the clearance of vascular *Candida*-derived β-glucan was rapid in the rabbit (half-life of 1.4–1.8 min). Interestingly, anti-β-glucan antibodies have been detected in humans (36) and other animals (37), and their presence may affect the clearance rate of β-glucan from the bloodstream. Our data on the clearance of injected β-1,6-glucan from the serum of mice indicate that the polysaccharide could be detected after 30 min of intravenous administration. To further understand the pharmacokinetics of naturally-released β-1,6-glucan and other β-glucans with a variety of composition ratios of β-1,3-glucan and β-1,6-glucan, future research will be required. Importantly, β-1,6-glucan could be detected with our probe *in vivo* from serum and several organs in the mouse model of systemic candidiasis. These preclinical data show promise for the potential development of a β-1,6-glucan–based detection system as a diagnostic modality for future clinical use.

In conclusion, we have found that a point mutation at amino acid position 321 (glutamic acid to glutamine) in the endo-β-1,6-glucanase Neg1 from *Neurospora crassa* promotes its function as a β-1,6-glucan–specific binding protein and provides a probe that has the potential for future diagnostic development. We are currently developing an immunoassay-based rapid glucan detection system with glucanase and magnetic beads, because the LAL test usually gets the results within 90 min, whereas the β-1,6-glucan ELISA requires 4 h. In addition, we are in the process of characterizing the structure of the natural form of the exopolysaccharide from various fungi using both the conventional β-1,3-d-glucan test and our β-1,6-glucan detection system.

## Experimental procedures

### Study approval

For the kinetic analysis of blood concentration of intravenously injected β-1,6-glucan, female ICR mice were purchased from Japan SLC (Shizuoka, Japan), housed in a specific pathogen-free (SPF) environment, and used at 7–10 weeks of age. The animal experimental protocol was approved by the Committee for Laboratory Animal Experiments at Tokyo University of Pharmacy and Life Sciences (P18–34), and the experiment was performed in accordance with the experiment guidelines provided by the Tokyo University of Pharmacy and Life Sciences. The mouse model of systemic candidiasis has been previously described (23). C57BL/6 WT and Cx3cr1-deficient mice were purchased from Taconic Farms and were maintained at the American Association for the Accreditation of Laboratory Animal Care-accredited animal facility at the NIAID (National Institutes of Health) under SPF conditions and housed in accordance with the procedures outlined in the Guide for the Care and Use of Laboratory Animals under the auspices of a protocol approved by the Animal Care and Use Committee of the NIAID (LCIM14E). Eight- to 12-week-old female mice were infected with *C. albicans* strain SC5314. Study protocols for *Candida* yeasts isolated from patients at the Kyorin University Hospital (895, 16-22) and the National Institutes of Health Clinical Center (11-I-0187) were approved by the Institutional Review Board committees at each study center. The study was performed in accordance with the Declaration of Helsinki.

### Materials

Gentiobiose, dimethylamine borane (DMAB), and 1,3-diaminopropane dihydrochloride (DAP-2HCl) were purchased from Tokyo Chemical Industry Co., Ltd. (Tokyo, Japan). Clear and white plates for the β-1,6-glucan ELISA were purchased from Greiner Bio-one (Frickenhausen, Germany). The peroxidase substrate, 3,3′,5,5′-tetramethylbenzidine (TMB) was purchased from KPL Inc. The soluble β-1,6-glucan polymer, pustulan from *Lasallia pustulata* was obtained from Calbiochem and InvivoGen. Laminarin (4, 38), mannan (39), barley BG (40), DMSO, and calcofluor white (CFW) were purchased from Sigma-Aldrich. Bovine serum albumin (BSA) was purchased from Sigma-Aldrich and Fisher. Sonifilans(SPG) (41) that have been used clinically as anticancer β-glucan in Japan were obtained from Kaken Pharmaceutical Co., Ltd. (Tokyo, Japan). We purchased SCL (42) from CarboMer, Inc., pullulan (43) from Pfanstiehl Laboratories Inc., and dextran T500 (44) from Pharmacia (Uppsala, Sweden). *Aureobasidium pullulans*–derived β-glucan, AP-FBG (45, 46), was gifted from ADEKA Corp. (Tokyo, Japan). BBG (47) was a gift from Oriental Yeast Co., Ltd. (Tokyo, Japan). Paramylon (48) and gentio-oligosaccharides (DP 2–6, mix) were purchased from Wako Pure Chemical Industries, Ltd. (Osaka, Japan). Structural characteristics of β-glucan and non-β-glucan used in this study is listed in Table S1.

### Fungal strains

*C. albicans* NBRC 1385 was a standard strain for *in vitro* culture; *C. albicans* NBRC 0692, 0759, 1060, 1061, 1393, 1397, 1594, and 1974; *N. crassa* NBRC 6068; and *Aspergillus niger* NBRC 6342 were all obtained from NBRC (Chiba, Japan). The Ca*big1*Δ strain *C. albicans* BIG104 that lacked CaBig1p, resulting in repression of β-1,6-glucan biosynthesis and its reconstituted strain *C. albicans* BIG105, was created in a previous study (49). Then 214 *Candida* yeasts (132 *C. albicans*; 35 *C. glabrata*; 15 *C. dubliniensis*; 11 *C. parapsilosis*; 11 *C. krusei*; 9 *C. tropicalis*; 1 *C. auris*) and 23 strains of *C. albicans* isolated from patients at the National Institutes of Health Clinical Center and Kyorin University Hospital, respectively, were tested for quantification of β-1,6-glucan in the culture supernatant *in vitro*. Ten additional isolates of *C. auris* (AR Bank numbers 0381 to 0390) were obtained from the FDA-CDC Antibiotic Resistance Isolate Bank and were also used for the *in vitro* culture test as mentioned above. For all mouse challenge experiments, *C. albicans* SC5314 was used.

### Preparation of polysaccharide fractions

The β-1,6-glucan islandican from *Penicillium islandicum* (50), *Agaricus brasiliensis*–derived AgCAS (51) that is rich in β-1,6-glucan, and the branched β-1,3-glucan SCG from *Sparassis crispa* (52, 53) were prepared as described previously. Solubilized β-glucan purified from cell wall, CSBG from *C. albicans* NBRC 1385 (54), ASBG from *A. niger* NBRC 6342 (55), and the exopolysaccharide CAWS released from *C. albicans* NBRC 1385 (17) were prepared according to previous reports. Chitin oligomers were prepared through acetone precipitation after hydrolysis in concentrated hydrochloric acid as described previously (56).

### Plasmid preparation

The mature form of recombinant endo-β-1,6-glucanase (Neg1, GH30_3, EC 3.2.1.75) was prepared as reported previously (19) with slight modifications. The β-1,6-glucanase coding gene (*neg1*) was amplified by PCR using PrimeSTAR Max DNA polymerase (Takara Bio Inc., Shiga, Japan) and primers pCold-IF-NEG1M-F and pCold-IF-NEG1-R with template cDNA prepared from *N. crassa* NBRC 6068. The PCR amplicon was purified and cloned into linearized pCold I DNA vector (Takara Bio Inc.) (1–300, 361–4407, amplified with primer sets pColdI-n361-F and pColdI-n300-R) using In-Fusion HD cloning kit (Clontech), then transformed into *E. coli* DH5α competent cells, cultured in LB broth containing ampicillin (100 μg/ml), and purified as Neg1–His_6_-tag fusion protein-expressing plasmid vector (pCold-Neg1). The point mutation at the catalytic domain (21) of Neg1, Glu-225 (acid/base), and/or Glu-321 (nucleophile) was induced using basic directional cloning methods. Linear vector and DNA inserts for glucanase variants were amplified by PCR using primer sets (vector for all variants, NEG1-Mu-F and NEG1-Mu-R; insert for E225Q, NEG1–225Q-F and NEG1–321E-R; insert for E321Q, NEG1–225E-F and NEG1–321Q-R; and insert for E225Q/E321Q, NEG1–225Q-F and NEG1–321Q-R) with pCold-Neg1 as a template plasmid. *Oplophorus gracilirostris*-derived low molecular weight luciferase, NanoLuc (57) (Nluc, 19 kDa)-fused Neg1–E321Q, was designed for the dot-blot assay. The DNA sequence encoding Nluc and stop codon removed Neg1–E321Q-encoding linear vector was amplified with primer sets (pCold-NL-IF-F and pCold-NL-IF-R, pColdI-n361-F and NEG1-FS-R, respectively) and joined by linker peptide (GGSGGGSGG) sequence. The protein-expressing plasmid vectors were prepared as described above, and DNA sequence was confirmed using BigDye Terminator version 3.1 cycle sequencing kit (Thermo Fisher Scientific) and an ABI3130xl DNA analyzer (Applied Biosystems). All sequences of primer sets used in this study are listed in Table S2.

### Preparation of endo-β-1,6-glucanase and its derivatives

SHuffle express competent *E. coli* cells (New England Biolabs) harboring each plasmid were cultured at 37 °C in LB broth with ampicillin (100 μg/ml) until the OD_600_ reached 0.4, and then isopropyl β-d-1-thiogalactopyranoside was added at final concentration of 0.01 mm and further incubated (180 rpm) at 15 °C for 24 h. The cells were collected and resuspended in PBS containing 0.2 mm phenylmethylsulfonyl fluoride and 1 mm DTT. After sonication was repeated three times for 30 s at 50 watts on ice, the insoluble fraction was removed by centrifugation (10,000 rpm, 20 min, 4 °C), and the supernatant was applied to TALON metal affinity resin (Clontech). After washing with sodium phosphate buffer (pH 7.0), His_6_-tagged Neg1 (52 kDa) and its derivatives were eluted by 150 mm imidazole-containing buffer and dialyzed against PBS (MWCO 3,500 Da), and protein concentration was measured by Pierce BCA protein assay kit (Thermo Fisher Scientific). The yields of recombinant glucanase and its derivatives are summarized in Table S3.

### Biotinylation of modified glucanase and pustulan

Neg1–E321Q–His (400 μg/ml) was biotinylated by mixing with a 5-fold molar excess of biotin-(AC_5_)_2_-N-hydroxysuccinimide ester (Osu) (Dojindo, Kumamoto, Japan) in PBS at room temperature for 1 h and then stored at 4 °C until use. For a quantitative measurement of glucanase affinity, biotinylation of pustulan at the reducing terminus was carried out by adding the primary amine moiety as described previously with modifications (58, 59). Briefly, 20 mg of DMAB dissolved in 100 μl of acetic acid at 80 °C was mixed with 5 mg (0.25 μmol) of pustulan and heated with 8 ml of DAP-2HCl solution (147 mg, dissolved in DMSO) at 80 °C for 1 h. Water was added to the reaction mixture and dialyzed (MWCO: 3,500 Da) against water three times; the buffer was changed to PBS and mixed with biotin-(AC_5_)_2_–N-hydroxysuccinimide ester (Osu) (0.5 μmol) at room temperature for 3 h. The reaction mixture was further dialyzed against water, and then the lyophilized biotin-labeled pustulan was reconstituted in PBS at 1 mg/ml by boiling.

### Measurement of (1→3)-β-d-glucan (LAL assay)

The concentration of β-1,3-d-glucan was analyzed by the chromogenic kinetic method, Fungitec G test MKII “Nissui” (Nissui Pharmaceutical Co., Ltd., Tokyo, Japan), with pachyman (60) as a standard glucan according to the manufacturer's instructions. The reaction of NaOH-diluted samples and the LAL reagent in the β-glucan–free 96-well microplates (Toxipet plate 96 F, Seikagaku Corp., Tokyo, Japan) was monitored by Wellreader MP-96 (Seikagaku Corp.) at 37 °C for 30 min.

### Verification of modified β-1,6-glucanase as the structure-specific probe

To verify the binding ability and its structure specificity of β-1,6-glucanase variants to glucans, direct and competitive ELISA-like assays were carried out. In brief, for direct ELISA, pustulan or laminarin (0–5,000 ng/ml) in 0.1 m sodium carbonate buffer (pH 9.5) was added to a 96-well clear plate and incubated at 4 °C. The next day, the plate was washed by PBS containing 0.05% Tween 20 (PBST) and blocked with 1% BSA/PBST (BPBST) by incubating for 1 h. Solid-phased glucans were reacted with recombinant modified Neg1 in BPBST (2 μg/ml) for 1 h and washed, and the HRP-conjugated anti-His tag antibody (BioLegend) was added to the plate. After 1 h, the plate was washed, and the binding of modified enzymes to solid-phase glucans was monitored using the peroxidase substrate TMB, and color development was stopped with 1 m phosphoric acid; the optical density was measured at 450 nm using a microplate reader (MTP450; Corona Electric, Ibaraki, Japan). For competitive ELISA, various glucans (20 and 100 μg/ml, final concentrations) were mixed with Neg1–E321Q–His (0.5 μg/ml, final concentration) and preincubated for 1 h. The pustulan (0.5 μg/ml)-coated 96-well clear plate was blocked, washed, and incubated with the above mixture of E321Q–His and glucan for 1 h. After washing, the binding of E321Q–His to the immobilized pustulan was assessed as described above.

### pH and thermal stability of modified β-1,6-glucanase

Stability of Neg1–E321Q was evaluated by ELISA under different conditions. Briefly, Neg1–E321Q–His (1 μg/ml) in PBS was pretreated at various temperatures (20–90 °C) for 5 min and cooled on ice, and an equal volume of BPBST was added. A 96-well clear plate was coated with pustulan (0.5 μg/ml), blocked by BPBST, and incubated with heat-treated Neg1–E321Q–His (0.5 μg/ml). To evaluate pH stability, untreated Neg1–E321Q–His (0.5 μg/ml) in 1% BSA-containing various pH environments with McIlvaine (pH 2.2–7.8) or modified Britton-Robinson (pH 4.0–11) buffer solutions (61, 62) was added to each well. Plate binding Neg1-E321Q-His was monitored by anti-His-Tag-mAb-HRP (0.5mg/ml, BioLegend) and HRP substrate TMB.

### Measurement of the affinities of β-1,6-glucanase derivatives

The binding affinity and the kinetics of modified Neg1 to the immobilized pustulan were monitored using the BLI biosensor (BLItz system; Pall ForteBio Inc.). The affinities of Neg1 variants were measured as follows. The streptavidin-coated biosensor chips were prehydrated, and the initial baseline was determined by incubating with assay buffer (PBS containing 0.1% BSA and 0.005% Tween 20) in the tube for 30 s. Biotin–pustulan (10 μg/ml) in 4 μl of assay buffer was loaded to sensor chips for 120 s and washed by assay buffer for 30 s (baseline). For the association assay, sensor chip and glucanase variants at 1 μg/ml (19.23 nm) in 4 μl of assay buffer were incubated for 120 s. Then, the assay buffer was applied to the sensor chip for 120 s to collect dissociation data. To calculate the association rate constant (*k_a_*), dissociation rate constant (*k_d_*), and equilibrium dissociation constants (*K_D_*) of E321Q–His, the data from 2-fold serially diluted seven concentrations (3.01, 6.01, 12.02, 24.04, 48.08, 96.15, and 192.3 nm) of test samples were collected. The data were analyzed using BLItz Pro software (Pall ForteBio Inc.). To assess the binding affinity of E321Q–His toward the unlabeled-soluble pustulan, ITC analysis was performed using an Affinity ITC instrument (TA Instruments). E321Q–His and pustulan were co-dialyzed twice against PBS. The average molecular mass (34,000 Da) of pustulan after dialysis was determined by HPLC with similar settings as described in a previous report (47). ITC was performed at 25 °C with 20 subsequent injections of 2.5 μl each. The data were analyzed using NanoAnalyze software (TA Instruments) as an independent model.

### β-1,6-Glucan ELISA for gentio-oligosaccharides

A 96-well clear plate was coated with gentio-oligosaccharides (0–100 μg/ml) or pustulan (0–20 μg/ml), blocked by BPBST, incubated with biotinylated-E321Q (2 μg/ml), and detected using streptavidin–HRP (BioLegend) and TMB. The reverse experiment was carried out to exclude the possibility that differential binding was due to the size of glucans in the plate. The pustulan (1 μg/ml)-coated 96-well clear plate was blocked and treated with mixture of biotinylated-E321Q (0.5 μg/ml, final concentration) and various concentrations of gentio-oligosaccharides (0–5,000 μg/ml, final concentrations) or pustulan (0–500 μg/ml, final concentrations). The biotinylated-E321Q binding to solid-phased pustulan was detected as described above.

### FACE

The reducing end of the oligosaccharide sample was labeled by fluorophore 8-aminonaphthalene-1,3,6-trisulfonic acid (ANTS; Invitrogen) and separated by PAGE (63, 64).

### Separation of hydrolyzed pustulan using HPLC

Pustulan (1 mg/ml) was hydrolyzed by boiling for 20 min with hydrochloric acid (0.1 n). Gel-permeation chromatography was performed to separate hydrolyzed pustulan on an HPLC system that consisted of a Waters 510 pump, a CTO-6A column oven (Shimadzu, Kyoto, Japan), and a Shodex GS-220 HQ column (7.5 × 300 mm, Showa Denko, Tokyo, Japan). The separation was performed at 60 °C using H_2_O as the eluent at a flow rate of 0.5 ml/min (56). Samples were fractionated every minute, lyophilized by centrifugal concentrator CC-105 (Tomy Seiko Co., Ltd., Tokyo, Japan), and dissolved in H_2_O to adjust the concentration. Oligosaccharide was labeled by ANTS and analyzed by FACE as described above.

### Dot-blot assay

To disclose the interaction between modified β-1,6-glucanase and trace amounts of oligosaccharide, ANTS-labeled acid-degraded pustulan in each fraction separated by HPLC was spotted (1.5 μl) onto a nylon membrane (0.45 μm, positively charged, Wako Pure Chemical Industries, Ltd.) with a border drawn by Western Sure Pen (LI-COR Biotechnology) in advance. The membrane was gently washed by ultrapure water containing 0.05% Tween 20, blocked with 1% casein sodium in washing buffer for 60 min, washed, and incubated with Neg1–E321Q–Nluc (2 μg/ml) in blocking buffer. After washing, membrane bound E321Q-Nluc was detected using Nano-Glo luciferase substrate (Promega, WI) in the chemiluminescent substrate (ImmunoStar; Wako Pure Chemical Industries, Ltd.). The images were scanned using a C-DiGit Blot Scanner (LI-COR Biotechnology).

### Binding of modified β-1,6-glucanase to C. albicans cell wall

Heat-killed *C. albicans* (HKCA) strain NBRC1385 grown in yeast extract, peptone, and dextrose (YPD) medium (48 h, yeast form) was repeatedly washed by PBS. Insoluble fungal body (300 μg/ml, final concentration) was mixed with Neg1–E321Q–His (0–5 μg/ml, final concentrations) in 50 μl of flow cytometry–staining buffer for 30 min. A competitive assay was used to prove the glucan-specific reaction, in which E321Q–His (5 μg/ml, final concentration) was pre-mixed with different concentrations of pustulan, laminarin, or mannan (0–100 μg/ml, final concentrations). Subsequently, cell wall–binding E321Q–His was detected using anti-His-tag mAb-biotin (MBL Co., Ltd., Aichi, Japan) and streptavidin–APC (BioLegend). FACS was performed using a BD Accuri C6 flow cytometer with BD CSampler software (BD Biosciences), and data were analyzed using FlowJo software (Tree Star Inc.). Formalin-killed *C. albicans* NBRC1385 grown in RPMI 1640 medium (Life Technologies Inc.) containing 10% heat-inactivated FBS (Equitech-Bio, TX) (24 h, hyphae form) was repeatedly washed by PBS, resuspended in 2% BSA/PBS, and incubated with Neg1–E321Q–His (5 μg/ml) and dectin-1–Fc (purified fusion protein of mouse dectin-1 carbohydrate-recognition domain and human IgG1–Fc domain expressed in silkworm, 2.5 μg/ml) as the β-1,3-glucan probe. After washing, *C. albicans* was further treated with anti-His-tag mAb–biotin. To detect cell wall mannan, 2 mg of concanavalin A (ConA) (Wako Pure Chemical Industries, Ltd.) was labeled with 100 μg of NHS–rhodamine (Thermo Fisher Scientific) in PBS at 4 °C (overnight), and Tris buffer was added to quench the reaction. Then, cells were washed and stained with streptavidin–APC (BioLegend), anti-human IgG1–Fc–FITC (BioLegend), ConA–rhodamine (10 μg/ml), and 5 μg/ml CFW for total chitin at 4 °C. The unbound reagents were removed by three PBS washes, and stained cells were mounted onto microscope slides with ProLong Diamond Antifade Mountant (Thermo Fisher Scientific). The image data were collected using a confocal laser scanning microscope (Olympus FV1000; Olympus, Tokyo, Japan).

### Quantification of β-1,6-glucan by a sandwich ELISA-like assay

A 96-well white plate was coated with Neg1–E321Q–His (2 μg/ml) by overnight incubation at 4 °C. The plate was washed with PBST and incubated for 1 h with BPBST. After washing, the diluted specimen and standard β-1,6-glucan (pustulan, InvivoGen) were added to the plate and incubated for 1 h at room temperature. Biotinized Neg1–E321Q–His (2 μg/ml) in BPBST was added to the washed plate and incubated for 1 h. The plate was then washed and treated with streptavidin–HRP (BioLegend or R&D Systems, MN) in BPBST for 20 min. After removing the unbound enzyme, the peroxidase substrate (SuperSignal ELISA femto substrate; Thermo Fisher Scientific) was added, and luminescence signals were measured using a microplate reader (GloMax; Promega or Spark; TECAN, Männedorf, Switzerland).

### In vitro culture of clinical Candida strains

Yeasts were inoculated onto YPD agar plates and cultured at room temperature for a few days, and then yeast colonies were suspended in formalin or sterile PBS. Representative clinically-isolated yeasts (10^5^ cells) in formalin were washed twice with FACS buffer and incubated with buffer only, Neg1–E321Q–His (8 μg/ml) or Neg1–E321Q–His plus pustulan (100 μg/ml). After washing, cells were further incubated in buffer only or PE-conjugated streptavidin (0.4 μg/ml, Miltenyi Biotec, Bergisch Gladbach, Germany) and then analyzed by cytometer (BD Fortessa and Diva software; BD Biosciences) and FlowJo software. Yeasts suspended in PBS were counted and further cultured in 10% FBS containing RPMI 1640 medium (10^5^ yeasts/ml) for 24–72 h at 37 °C. After centrifugation at 1,800 × *g* for 10 min at 4 °C, fungus-free supernatants were collected and kept frozen at −20 °C until used for β-glucan test. The remaining fungal body was fixed in formalin and photographed with EVOS FL Cell Imaging System (Thermo Fisher Scientific) to prove the proliferation. The culture supernatant was boiled for 5 min before use for the β-glucan test.

### Murine model of systemic candidiasis

Mouse experiments were performed as described previously (24). Briefly, the *C. albicans* strain SC5314 was grown in YPD medium containing penicillin and streptomycin (Mediatech Inc.) in a shaking incubator at 30 °C. Cells were centrifuged, washed in PBS, counted, and injected (10^5^ yeast cells) into C57BL/6 mice via the lateral tail vein. The serum, kidney, spleen, liver, and brain were harvested before infection and at days 3, 6, and 9 after infection, and the organs were homogenized using a tissue homogenizer (Omni International, Inc.) into 1.5 ml of PBS with 0.5% Tween 20 and a protease inhibitor mixture (Roche Applied Science, Upper Bavaria, Germany) and centrifuged at 15,682 × *g* for 10 min at 4 °C. The clarified supernatants and serum were frozen at −80 °C until use.

### Pretreatment of specimen for quantification of β-1,6-glucan

The appropriate volume of PBS (equal volume for serum, 4× volume for organs) was added to the serum and supernatants of organs from *Candida*-infected mice, and they were boiled for 5 min, mixed, and centrifuged at 14,000 rpm for 10 min at 4 °C before use for ELISA.

### Statistical analyses

GraphPad Prism 7.0 (GraphPad Software) was used for all statistical analyses. Normal distributions of the data were analyzed by Shapiro-Wilk or Kolmogorov-Smirnov tests. Significant differences were analyzed by two-tailed unpaired *t* test or Mann-Whitney *U* test as appropriate according to the results of the distribution tests. Wilcoxon signed-rank test was used for the kinetic analysis of the *in vitro* culture of *C. glabrata. p* values less than 0.05 were considered significant.

## Author contributions

D. Y. conceptualization; D. Y., H. O., T. U., F. O., M. S. L., and N. O. resources; D. Y. data curation; D. Y. formal analysis; D. Y., M. S. L., and N. O. supervision; D. Y., F. O., M. S. L., and N. O. funding acquisition; D. Y., K. T., M. K., M. S., and T. U. validation; D. Y., K. T., M. K., M. S., and T. U. investigation; D. Y., K. T., and M. K. visualization; D. Y., H. O., T. U., F. O., M. S. L., and N. O. methodology; D. Y. and M. S. L. writing-original draft; D. Y., M. S. L., and N. O. project administration; D. Y., M. K., H. O., T. U., F. O., M. S. L., and N. O. writing-review and editing.

## Supplementary Material

Supporting Information

## Footnotes

2 The abbreviations used are:

LAL: *Limulus* amebocyte lysate
ANTS: 8-aminonaphthalene-1,3,6-trisulfonic acid
BLI: bio-layer interferometry
CFW: calcofluor white
ConA: concanavalin A
DAP-2HCl: 1,3-diaminopropane dihydrochloride
DMAB: dimethylamine borane
FACE: fluorophore-assisted carbohydrate electrophoresis
FBS: fetal bovine serum
HRP: horseradish peroxidase
ITC: isothermal titration calorimetry
SPF: specific pathogen-free
TMB: 3,3′,5,5′-tetramethylbenzidine
HKCA: heat-killed yeast form of *C. albicans*
PE: phycoerythrin
BG: β-glucan
GH: glycoside hydrolase
SPG: schizophyllan
SCG: *Sparassis* β-glucan
SCL: scleroglucan
BBG: bakers' yeast-derived β-glucan
oligo: oligosaccharide
DP: degree of polymerization
NBRC: NITE Biological Resource Center.
