# Supplementary material for: Development of a novel β-1,6-glucan–specific detection system using functionally-modified recombinant endo-β-1,6-glucanase
Source: J Biol Chem. 2020 Mar 4;295(16):5362–76. doi: 10.1074/jbc.RA119.011851 (PMC7170528; doi:10.1074/jbc.RA119.011851)
Supplement: Supporting Information [file supp_295_16_5362__index.html]

Development of a novel β-1,6-glucan-specific detection system using functionally modified recombinant endo-β-1,6-glucanase — Detection and quantification of β-1,6-glucan — Development of a novel β-1,6-glucan–specific detection system using functionally-modified recombinant endo-β-1,6-glucanase — Detection and quantification of β-1,6-glucan — Supporting Information 

# Development of a novel β-1,6-glucan–specific detection system using functionally-modified recombinant endo-β-1,6-glucanase

## Supporting Information

- Supporting Information (to be published online) - Tables S1-S3 Figures S1-S6
