# Supplementary material for: Development of a novel β-1,6-glucan–specific detection system using functionally-modified recombinant endo-β-1,6-glucanase
Source: J Biol Chem. 2020 Mar 4;295(16):5362–76. doi: 10.1074/jbc.RA119.011851 (PMC7170528; doi:10.1074/jbc.RA119.011851)
Supplement: Supporting Information [file supp_RA119.011851_157050_2_supp_483645_q6f33c.pdf]

Supporting Information

Development of a novel β-1,6-glucan-specific detection system using functionally modified recombinant endo-β-1,6-glucanase

Daisuke Yamanaka[1, 2*], Kazushiro Takatsu[1], Masahiro Kimura[3, 4], Muthulekha Swamydas[2], Hiroaki Ohnishi[5], Takashi Umeyama[6], Fumitaka Oyama[3], Michail S. Lionakis[2] and Naohito Ohno[1]

[1]*Laboratory for Immunopharmacology of Microbial Products, School of Pharmacy, Tokyo University of Pharmacy and Life Sciences, Hachioji, Tokyo, Japan*
[2]*Fungal Pathogenesis Section, Laboratory of Clinical Immunology & Microbiology (LCIM), National Institute of Allergy and Infectious Diseases (NIAID), National Institutes of Health (NIH), Bethesda, Maryland, USA*
[3]*Department of Chemistry and Life Science, Kogakuin University, Hachioji, Tokyo, Japan*
[4]*Research Fellow of Japan Society for the Promotion of Science (DC2), Koujimachi, Chiyoda-ku, Tokyo 102-0083, Japan*
[5]*Department of Laboratory Medicine, Kyorin University School of Medicine, Mitaka, Tokyo, Japan*
[6]*Department of Chemotherapy and Mycoses, National Institute of Infectious Diseases, Shinjuku-ku, Tokyo, Japan*
*To whom correspondence should be addressed: Daisuke Yamanaka, Ph.D.; Laboratory for Immunopharmacology of Microbial Products, School of Pharmacy, Tokyo University of Pharmacy and Life Sciences, 1432-1 Horinouchi, Hachioji, Tokyo 192-0392, Japan; Tel: +81-426-76-5570; Fax: +81-426-76-5570; E-mail: ymnkd@toyaku.ac.jp

Table of Contents

Supplementary Methods
Table S1. Glucans used in this study.
Table S2. Primer sequences used in this study.
Table S3. The yield of recombinant proteins.
Figure S1. Reactivity of the LAL reagent with representative β-glucans.
Figure S2. ^{1}H, ^{13}C-HSQC spectra of representative β-glucans used in this study.
Figure S3. SDS-PAGE and Western blot analyses of purified recombinant Neg1-derivatives.
Figure S4. Glycolytic activity of Neg1-derivatives.
Figure S5. Competition of HRP substrates for the standard curve of the quantitative sandwich ELISA.
Figure S6. β-1,6-glucan is produced during *Candida* systemic infection in *Cx3cr1*$^{-/-}$ mice.

Supplementary Methods

Materials

Somogyi's copper reagent, Nelson's arsenomolybdate reagent and Silver stain II kit were purchased from Wako Pure Chemical Industries, Ltd., and Coomassie Brilliant Blue (Rapid CBB KANTO, CBB-R250) was from Kanto Kagaku Co. (Tokyo, Japan). D_2O (deuteration degree, min. 99.9%) and Me_2SO-d_6 (99.8%) were purchased from Merck (Darmstadt, Germany).

NMR spectroscopy

NMR analysis of glucan samples was performed as previously reported (1). Briefly, the glucan sample was dissolved in D_2O and dried exchangeable protons were removed by repeated lyophilization. All the spectra were recorded in a mixed solvent, Me_2SO-d_6/D_2O (6:1) (10 mg/ml), at 50°C on a Bruker Avance 600 spectrometer. 2D ^{1}H, ^{13}C-heteronuclear single quantum coherence (HSQC) was performed by the Bruker standard pulse sequence and data was analyzed using Ramo 2D software (Sankyo shuppan Co., Ltd., Tokyo, Japan). In all the experiments, a squared cosine window function was applied for both dimensions.

SDS-PAGE and immunoblotting

Purified recombinant proteins used in this study were separated by 11% polyacrylamide gel electrophoresis and protein bands were visualized by silver nitrate or Coomassie Blue staining. The separated proteins were also transferred onto an amersham hybond ECL nitrocellulose membrane (GE Healthcare, IL, USA). After blocking with 0.05% Tween 20/Tris-buffered saline containing 0.5% skim milk (Cell Signaling Technology, MA, USA), membranes were incubated with anti-His-tag mAb-Biotin, detected using streptavidin-HRP (BioLegend) and HRP substrate (ImmunoStar). The images were scanned using a C-DiGit Blot Scanner and Image Studio Digits 4.0 Software (LI-COR Biotechnology).

Measurement of β-glucanase activity

The enzymatic activity was evaluated by visual analysis for oligosaccharide production and quantification of the reducing end of oligosaccharides. Pustulan (1 mg/ml) and recombinant Neg1 (1 µg/ml) were mixed with in 50 mM sodium acetate buffer (pH 6.0), incubated for 0, 6 or 24 h at 37°C and then the reaction mixture was boiled for 10 min to stop the enzyme reaction. The reducing ends of the reaction mixture and standard glucan (glucose, gentiobiose and gentio-oligosaccharides) were labeled by ANTS and analyzed by FACE. The increase in the level of the reducing sugar was measured by a slightly modified Nelson-Somogyi method (assay range: 100–800 µg/ml for glucose standard) (2, 3). Recombinant Neg1 and variants (1 or 10 µg/ml) were mixed with pustulan (1 mg/ml) in the same buffer, incubated for 1 h at 37°C and then, reaction mixture was boiled for 10 min. The reaction mixture or glucose solution for standard (5 µl) was mixed with 5 µl of Somogyi's reagent, treated for 20 min at 98°C, and 5 µl of Nelson's reagent was added to the cooled sample. After 30 min, 110 µl of water was added and the optical density was measured at 630 nm using a standard 96 well plate and a plate reader (MTP450). We further examined whether the glucanase activity of E321Q-His returned over time. The following experiments used the engineered probe that was stored for at least two years after purification. Kinetic parameters were determined using a natural substrate (pustulan) and the results were analyzed using GraphPad Prism software. Pustulan (0.25–10 mg/ml) was incubated at 37°C for 10 min with enzymes (50 µg/ml) in 50 mM sodium acetate buffer (pH 5.0). After boiling, the initial rates of increase in the reducing sugar levels were measured by the Nelson-Somogyi method. Furthermore, pustulan (10 mg/ml in PBS) was mixed with enzymes (50 µg/ml) at 37°C and the reaction time was extended to 48 h. After boiling, the amount of glucose or reducing sugar in the

supernatant were monitored using the glucose-glo assay kit (Promega, assay range: 3.1–50,000 nM for glucose standard) and the Nelson-Somogyi method. The hydrolytic activity against the synthetic substrate, *p*-nitrophenyl-β-D-glucopyranoside (Nacalai tesque, Kyoto, Japan), was also monitored. An enzyme (50 μg/ml) and substrate (20 mM) mixture in 50 mM sodium acetate buffer (pH 5.0) was incubated at 37°C for 16 h, after which the released *p*-nitrophenol was measured at 405 nm with standard *p*-nitrophenol (Wako Pure Chemical Industries, Ltd.).

1. Yamanaka, D., Tada, R., Adachi, Y., Ishibashi, K., Motoi, M., Iwakura, Y., and Ohno, N. (2012) *Agaricus brasiliensis*-derived β-glucans exert immunoenhancing effects via a dectin-1-dependent pathway. *International Immunopharmacology* **14**, 311-319
2. Somogyi, M. (1952) Notes on sugar determination. *Journal of Biological Chemistry* **195**, 19-23
3. Nelson, N. (1944) A photometric adaptation of the Somogyi method for the determination of glucose. *Journal of Biological Chemistry* **153**, 375-380

Table S1. Glucans used in this study.

**Glucan**	**Source**	**Structure [Size]**
Pustulan	*Lasallia pustulata*	β-1,6-glucan (with slight β-1,3-glucan)
Islandican	*Penicillium islandicum*	β-1,6-glucan
Pachyman	*Wolfiporia extensa*	β-1,6-/β-1,3-glucan
Laminarin	*Laminaria digitata*	mono-β-1,6-/β-1,3-glucan [5,850 Da]
Paramylon	*Euglena gracilis*	linear β-1,3-glucan [212,000 Da]
SPG	*Schizophyllum commun*	mono-β-1,6-/β-1,3-glucan [450,000 Da]
AgCAS	*Agaricus brasiliensis*	β-1,3-/β-1,6-glucan, mannoprotein
SCG	*Sparassis crispa*	β-1,6-/β-1,3-glucan
SCL	*Sclerotium rolfsii*	β-1,6-/β-1,3-glucan [1,400,000 Da]
BBG	Baker's yeast	β-1,6-/β-1,3-glucan, mannan
AP-FBG	*Aureobasidium pullulans*	β-1,6-/β-1,3-glucan [300,000 Da]
Barley BG	Barley	β-1,3-/β-1,4-glucan [>23,100 Da]
Pullulan	*Aureobasidium pullulans*	α-1,4-/α-1,6-glucan [100,000 Da]
Dextran	*Leuconostoc mesenteroides*	α-1,4-/α-1,6-glucan [500,000 Da]
Chitin oligomers	Shrimp shell chitin	β-1,4-*N*-acetyl-D-glucosamine unit [DP ≥6]
Mannan	*Saccharomyces cerevisiae*	α-1,6-/α-1,2-, α-1,3-mannan [14,500 Da]
CAWS	*Candida albicans*	mannoprotein, β-1,3-/β-1,6-glucan complex
CSBG	*Candida albicans*	β-1,6-/β-1,3-glucan
ASBG	*Aspergillus niger*	β-1,6-/β-1,3-glucan
Gentio-oligo	–	β-1,6-glucan oligomer [DP 2–6]
Gentiobiose	–	β-1,6-glucan dimer [DP 2]

Table S2. Primer sequences used in this study.

**Primer**	**Sequence**
pCold-IF-NEG1M-F	5’-CATATCGAAGGTAGGGCGATCCAACCCCAA-3’
pCold-IF-NEG1-R	5’-AGCAGAGATTACCTATTACGCCCCTGCAGCCGG-3’
pColdI-n361-F	5’-TAGGTAATCTCTGCTTAAAAGCACAG-3’
pColdI-n300-R	5’-CCTACCTTCGATATGATGATGATGAT-3’
NEG1-Mu-R	5’-GTTTTGGATGGTGATGGCGT-3’
NEG1-Mu-F	5’-TGCTGGACGTCAGCAAGGCA-3’
NEG1-225E-F	5’-ATCACCATCCAAAACGAACCGCTCAACTCG-3’
NEG1-225Q-F	5’-ATCACCATCCAAAACCAACCGCTCAACTCG-3’
NEG1-321E-R	5’-TGCTGACGTCCAGCACTCAGTCATGTACTGCTC-3’
NEG1-321Q-R	5’-TGCTGACGTCCAGCACTGAGTCATGTACTGCTC-3’
pCold-NL-IF-F	5’-GGCGGCAGCGGCGGCGGCAGCGGCGGCGTCTTCACACTCGAAGATTTC-3’
pCold-NL-IF-R	5’-AGCAGAGATTACCTATTACGCCAGAATGCGTTC-3’
NEG1-FS-R	5’-GCCGCCGCTGCCGCCGCCGCTGCCGCCCGCCCCTGCAGCCGG-3’

Table S3. The yield of recombinant proteins.

**Protein**	**Molecular weight**	**Yield of purified protein (mg/200 ml)**
His_6-taged Neg1	52 kDa	0.18
His_6-taged Neg1-E225Q	52 kDa	1.0
His_6-taged Neg1-E321Q	52 kDa	0.51 ± 0.1 (*n* = 4)
His_6-taged Neg1-E225Q/E321Q	52 kDa	0.94
His_6-taged Neg1-E321Q-Nluc	71 kDa	0.83

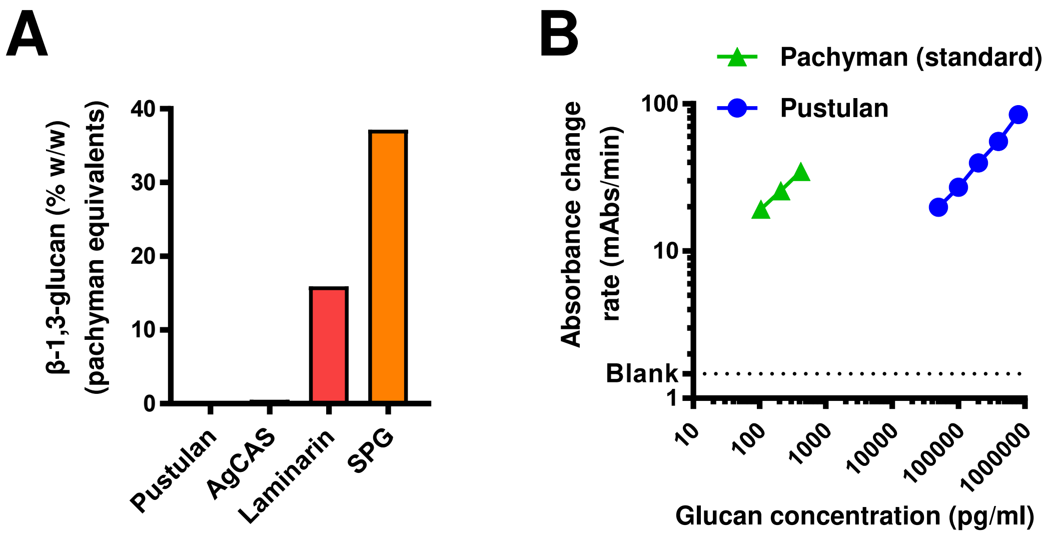

Figure S1. Reactivity of the LAL reagent with representative β-glucans. (A) Reactivity of the commercial LAL G test to pustulan, AgCAS, laminarin and SPG. The Factor G responsible β-1,3-D-glucan moiety of each glucan was indicated as pachyman equivalents (% w/w). (B) Comparison of reactivity of different concentrations of pachyman (100, 200 and 400 pg/ml) and pustulan (50, 100, 200, 400 and 800 ng/ml) in the LAL G test. 100 pg/ml of pachyman (19.3 mAbs/min) and 50 ng/ml of pustulan (19.9 mAbs/min) showed similar absorbance change rates.

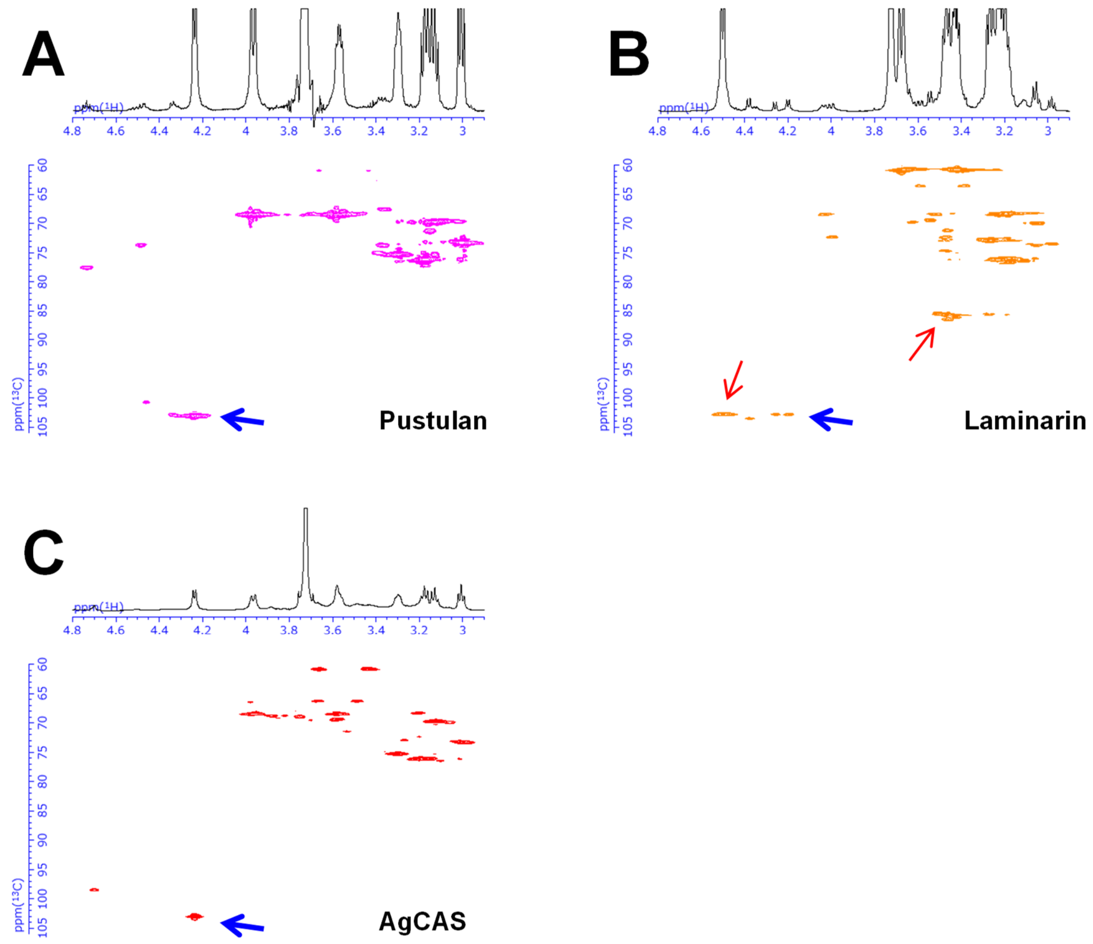

Figure S2. ^{1}H, ^{13}C-HSQC spectra of representative β-glucans used in this study. 2D ^{1}H, ^{13}C-HSQC spectra of (A) pustulan, (B) laminarin and (C) AgCAS were recorded in a mixed solvent of Me_2SO-d_6/D_2O (6:1) at 50°C using Bruker Avance 600 spectrometer. Arrows indicate the signals corresponding to β-1,3-glucosyl linkages (red) and β-1,6-glucosyl linkages (blue). Chemical shifts are reported in ppm relative to Me_2SO-d_6 as an internal standard.

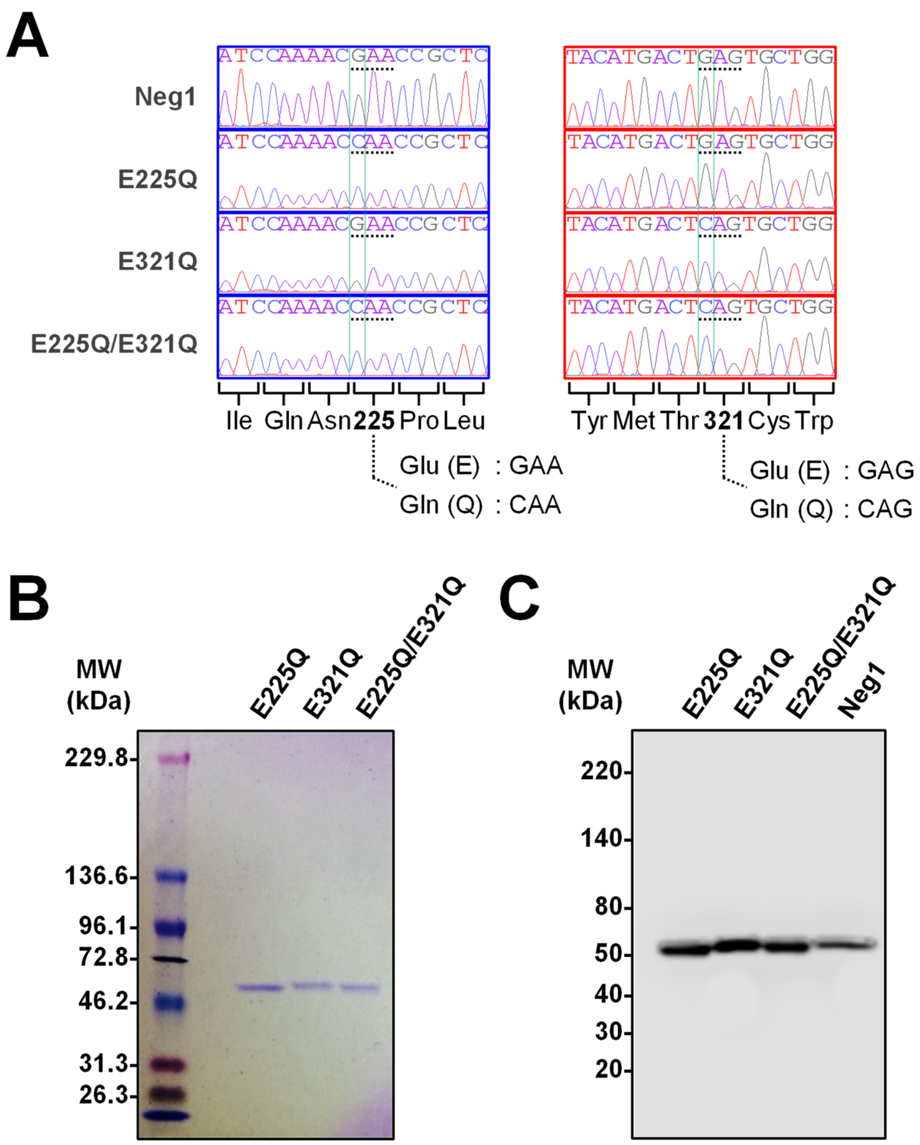

Figure S3. SDS-PAGE and Western blot analyses of purified recombinant Neg1-derivatives. (A) DNA-sequencing data around the catalytic domain at amino acid positions 225 and 321 of DNA plasmids encoding Neg1-derivatives. (B) SDS-PAGE image of purified Neg1-derivatives. Proteins were separated by 11% polyacrylamide gel and stained with Coomassie Brilliant Blue. (C) Immunoblot image of Neg1-derivatives. Proteins transferred to nitrocellulose membrane were detected by anti-His-tag mAb-Biotin and streptavidin-HRP.

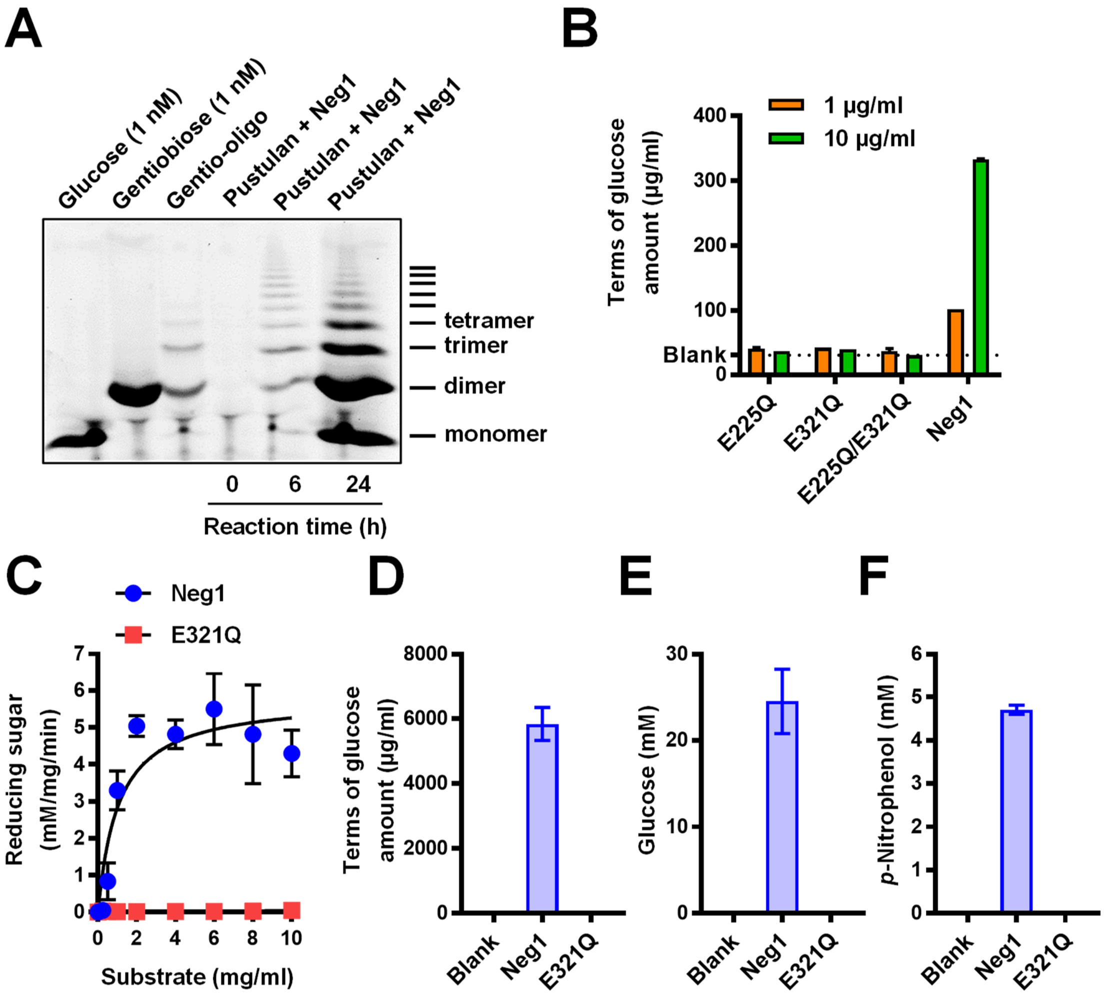

Figure S4. Glucan hydrolase activity of Neg1-derivatives. (A) Detection of oligosaccharides using FACE. Pustulan (1 mg/ml) was degraded by Neg1 (1 μg/ml) for 0, 6 or 24 h at 37°C and labeled by ANTS. Glucose, gentiobiose and gentio-oligo are shown in the left margin as standards. (B) Quantification of the amount of increased reducing end. Pustulan (1 mg/ml) was treated with Neg1-derivatives (1 or 10 μg/ml) for 1 h at 37°C. Nelson-Somogyi assay was conducted with blank, reaction mixtures and glucose as the standard. (C) Change initial rates against substrate concentration for Neg1 and Neg1-E321Q stored for a long period of time. Initial rates of increase in the level of reducing sugars in the reaction mixture of enzymes and pustulan (0.25–10 mg/ml) were measured by the Nelson-Somogyi assay. Kinetic parameters, K_m were calculated a Michaelis-Menten kinetic model. (D to F) Glucan hydrolase activity of Neg1-E321Q stored for a long period of time. The amount of (D) increase in the reducing sugar level and (E) released glucose after reaction with pustulan (10 mg/ml) for 48 h, and (F) the *p*-nitrophenol released after reaction with *p*-nitrophenyl-β-D-glucopyranoside (20 mM) for 16 h. Data are presented as mean ± SD of values in duplicate (B) or triplicate (D to F), or (C) from three independent experiments.

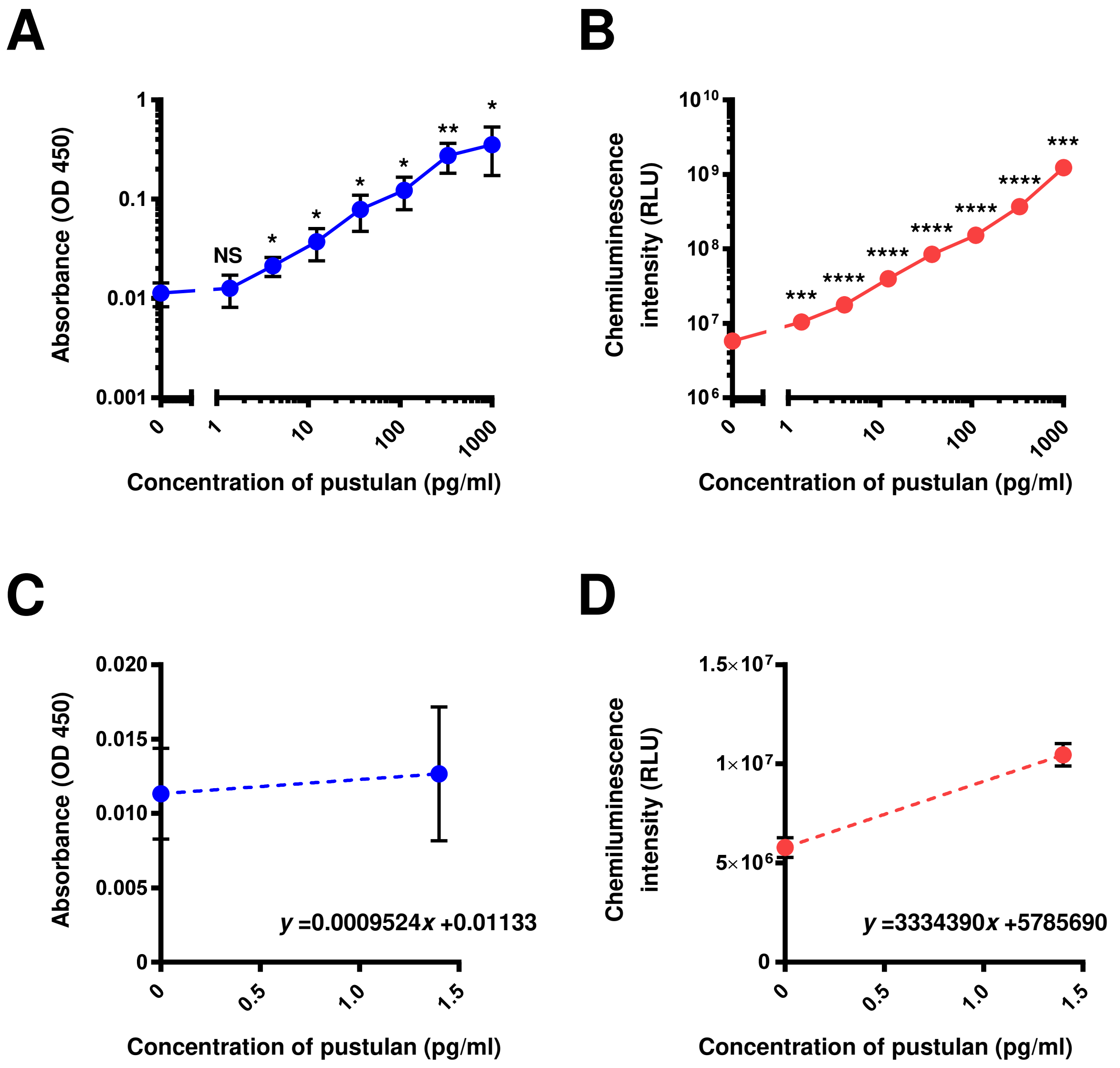

Figure S5. Competition of HRP substrates for the standard curve of the quantitative sandwich ELISA. Three-fold serial dilutions of pustulan (1.4 to 1,000 pg/ml) and blank were applied to the sandwich ELISA-like assay using Neg1-E321Q. The ELISA was conducted with the same conditions on the 96-well clear or white plates. (A, C) For the colorimetric method, clear plate and TMB substrate were used. (B, D) The white plate and SuperSignal femto substrate were used for the chemiluminescent method. (C, D) Regression equation was calculated from the fitting line (linear; y=ax+b) between blank and the lowest concentration of pustulan. The limit of detection (LoD) and limit of quantification (LoQ) were determined by basic calculation procedures; 3.3 × SD of replicates of blank/slope (a) for LoD, 10 × SD of replicates of blank/slope (a) for LoQ. The resultant LoD and LoQ of pustulan from the colorimetric and chemiluminescent methods were determined to be 10.6 and 32.1 pg/ml (TMB), and 0.5 and 1.5 pg/ml (SuperSignal), respectively. Data are presented as mean ± SD of values in triplicate. Significant differences relative to blank: *$p < 0.05$, **$p < 0.01$, ***$p < 0.001$, ****$p < 0.0001$, NS; not significant.

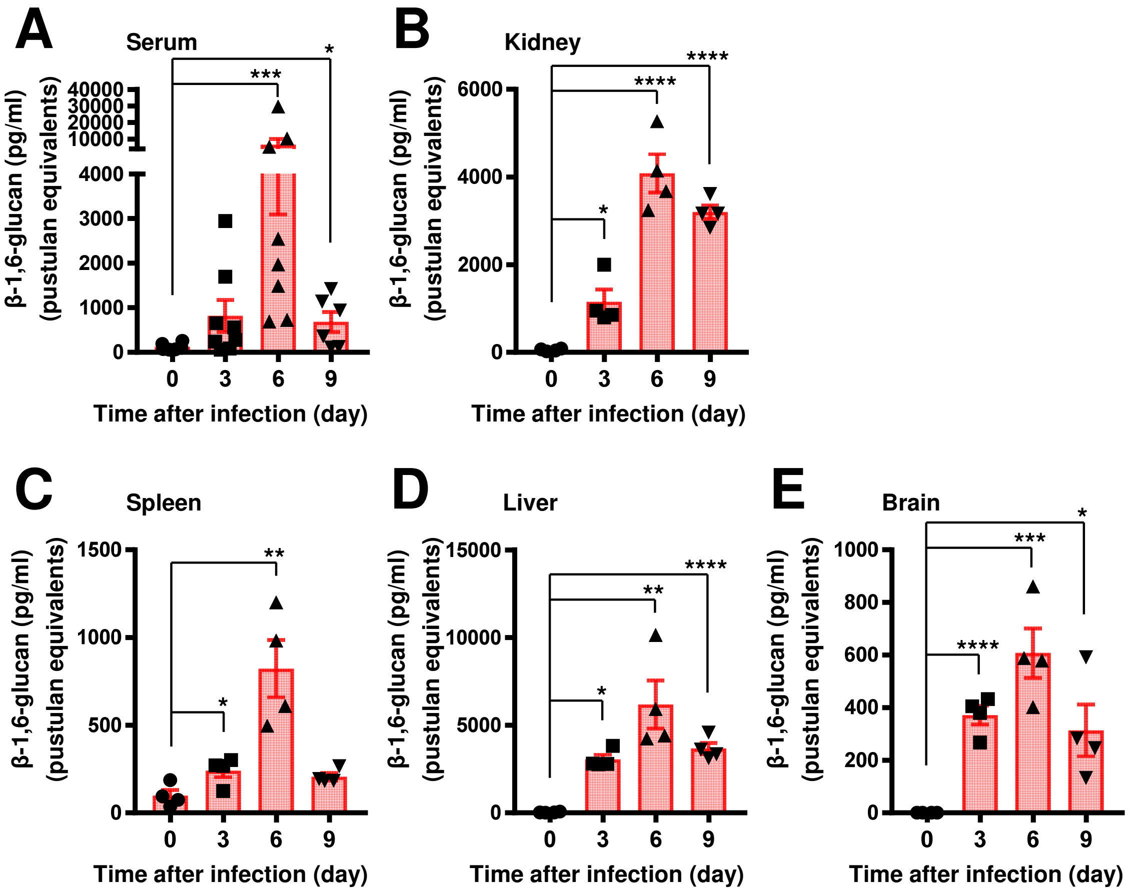

Figure S6. β-1,6-glucan is produced during *Candida* systemic infection in *Cx3cr1*$^{-/-}$ mice. Concentrations of β-1,6-glucan in (A) serum, (B) kidney, (C) spleen, (D) liver and (E) brain harvested from Cx3cr1-deficient mice on days 0, 3, 6 and 9 after *C. albicans* intravenous injection were measured by sandwich ELISA-like assay using Neg1-E321Q with pustulan as the standard of β-1,6-glucan. Data are presented as mean ± SEM (n = 6–8 for serum, n = 4 for organs). Significant differences relative to day 0: *p < 0.05, **p < 0.01, ***p < 0.001, ****p < 0.0001.
